# Norepinephrine-stimulated HSCs secrete sFRP1 to promote HCC progression following chronic stress via augmentation of a Wnt16B/β-catenin positive feedback loop

**DOI:** 10.1186/s13046-020-01568-0

**Published:** 2020-04-15

**Authors:** Xia-Hui Lin, Hua-Hua Liu, Shu-Jung Hsu, Rui Zhang, Jie Chen, Jun Chen, Dong-Mei Gao, Jie-Feng Cui, Zheng-Gang Ren, Rong-Xin Chen

**Affiliations:** Liver Cancer Institute, Zhongshan Hospital, Fudan University and Key Laboratory of Carcinogenesis and Cancer Invasion, Ministry of Education, Shanghai, 200032 P.R. China

**Keywords:** Chronic stress, Norepinephrine, Hepatic stellate cells, Hepatocellular carcinoma, SFRP1

## Abstract

**Background:**

Sustained adrenergic signaling secondary to chronic stress promotes cancer progression; however, the underlying mechanisms for this phenomenon remain unclear. Hepatocellular carcinoma (HCC) frequently develops within fibrotic livers rich in activated hepatic stellate cells (HSCs). Here, we examined whether the stress hormone norepinephrine (NE) could accelerate HCC progression by modulating HSCs activities.

**Methods:**

HCC cells were exposed to conditioned medium (CM) from NE-stimulated HSCs. The changes in cell migration and invasion, epithelial-mesenchymal transition, parameters of cell proliferation, and levels of cancer stem cell markers were analyzed. Moreover, the in vivo tumor progression of HCC cells inoculated with HSCs was studied in nude mice subjected to chronic restraint stress.

**Results:**

CM from NE-treated HSCs significantly promoted cell migration and invasion, epithelial-mesenchymal transition (EMT), and expression of cell proliferation-related genes and cancer stem cell markers in HCC cells. These pro-tumoral effects were markedly reduced by depleting secreted frizzled related protein 1 (sFRP1) in CM. The pro-tumoral functions of sFRP1 were dependent on β-catenin activation, and sFRP1 augmented the binding of Wnt16B to its receptor FZD7, resulting in enhanced β-catenin activity. Additionally, sFRP1 enhanced Wnt16B expression, reinforcing an autocrine feedback loop of Wnt16B/β-catenin signaling. The expression of sFRP1 in HSCs promoted HCC progression in an in vivo model under chronic restraint stress, which was largely attenuated by sFRP1 knockdown.

**Conclusions:**

We identify a new mechanism by which chronic stress promotes HCC progression. In this model, NE activates HSCs to secrete sFRP1, which cooperates with a Wnt16B/β-catenin positive feedback loop. Our findings have therapeutic implications for the treatment of chronic stress-promoted HCC progression.

## Background

Emerging evidence suggests that chronic stress in patients, including fear, anxiety, and depression, can promote cancer progression by inducing overactivity of the sympathetic nervous system (SNS) [1–4]. Chronic stress has been shown to increase the production of stress hormones such as norepinephrine (NE) and epinephrine from the SNS, enhancing tumor growth, invasion, and metastases in multiple malignancies [5–7]. However, the molecular mechanisms underlying the effects of chronic stress on cancer promotion are not fully understood.

Hepatocellular carcinoma (HCC) ranks fourth among cancer-related deaths worldwide [8]. HCC often arises on a background of inflamed or fibrotic livers rich in activated hepatic stellate cells (HSCs) [9–11]. As major sources of extracellular matrix (ECM) deposition, activated HSCs can profoundly alter the tumor microenvironment [11–13], and interactions between HCC and activated HSCs greatly influence HCC onset and progression [14–17]. In addition, it has been reported that hyperactive SNS contributes to the stress-induced aggravation of many liver diseases through its catecholamine neurotransmitters (including NE) [2, 18, 19]. Further, several neuronal genes were found to be highly expressed in HSCs, including adrenergic receptors [20–26]. This prompted us to hypothesize that chronic stress could affect HCC progression through stress hormone-modulating HSC activities.

In this study, we showed the crosstalk between chronic stress and HCC progression through NE-mediated activation of HSCs. Our observations included the following: (i) conditioned medium from NE-stimulated HSCs significantly enhanced the malignant phenotype of HCC cells; (ii) secreted frizzled-related protein 1 (sFRP1), a regulator of Wnt signaling, played a key role in the stress response of HSCs to NE and mediated the pro-tumoral effects of HSCs on HCC cells; (iii) sFRP1 enhanced Wnt16B expression to augment an autocrine feedback loop of Wnt16B/β-catenin signaling in HCC cells; (iv) expression of sFRP1 in HSCs accelerated HCC progression in an in vivo model under chronic restraint stress, which was significantly thwarted by the knockdown sFRP1. This study has therapeutic implications for the treatment of chronic stress-driven HCC progression.

## Materials and methods

### Cell lines and culture conditions

The human liver cell L02 (Cell Bank of the Chinese Academy of Sciences, Shanghai, China), human HCC cell lines PLC/PRF/5, HepG2 and Hep3B (ATCC, USA), SMMC7721 (Cell Bank of the Chinese Academy of Sciences, Shanghai, China), MHCC97H and HCCLM3 (Liver Cancer Institute, Fudan University, Shanghai, China), and Huh7 (Japanese Cancer Research Resources Bank) were propagated in the corresponding culture medium RPMI-1640 or Dulbecco’s modified Eagle’s medium (DMEM) with 10% fetal bovine serum (FBS, Gibco) and 1% penicillin-streptomycin (Invitrogen). Human hepatic stellate cell line LX-2 (a gift from SL. Friedman, Mount Sinai, New York) were grown in DMEM containing 2% FBS. Primary human hepatic stellate cells (pHSCs) (ScienCell, USA) were maintained in the provided complete stellate cell medium. All cell cultures were conducted at 37 °C in a thermostatic incubator containing 5% CO_2_.

### Norepinephrine (NE) treatment

For NE treatments, LX-2 cells were seeded in 6-well plates (2 × 10^5^ cells/well) (Corning Inc., USA) for 8 h to allow the cells to adherent, and then treated with increasing concentrations of NE (0.1, 1, 5 and 10 μM) (Sigma-Aldrich Co., St. Louis, MO) for 24 h.

Conditioned media (CM) collected from NE-treated HSCs were used to cultivate HCC cells. In brief, LX2 cells or sRFP1 knockdown LX2 cells (LX-2^shRNA sFRP1^) were treated with 10 μM NE for 24 h. The next day, the medium was exchanged with fresh DMEM supplemented with 2% FBS without NE, and the cells were cultured for another 24 h. The supernatant of cells was harvested, filtered and centrifuged with 3000 rpm for 15 min to remove debris. For individual experiments, HCCs were incubated with CM collected from NE-treated HSCs.

### Transfection of Lentiviral vectors with shRNA for sFRP1

LX2 cells with stable sFRP1 knockdown were generated using lentivirus-mediated knockdown of sFRP1. Lentiviral vectors encoding shRNA targeting sFRP1 or scramble (negative control) were purchased from the GENECHEM gene company, Shanghai, China. LX2 cells were transfected by lentivirus particles (multiplicity of infections, MOI =5) and then selected in culture medium with 2 μg/ml puromycin. The efficiency of gene silencing was assayed by quantitative reverse-transcription polymerase chain reaction (qRT-PCR) and Western blot.

### Quantitative reverse-transcription polymerase chain reaction (qRT-PCR)

Total RNA was extracted from the cells or tissues using TRIzol reagent (Invitrogen, USA), Then, synthesis of cDNA reaction was carried out with 2 μg of total RNA using a PrimeScript RT Reagent Kit (Takara, Japan) following the manufacturer’s instructions. Subsequently, PCR amplification was done on cDNA using Maxinma SYBR Green qPCR Master Mix (Thermo Scientific). Quantification of target genes was performed with the 2^−ΔΔCt^ method using glyceraldehyde-3-phosphate dehydrogenase (GAPDH) or β-actin for normalization. Melting curve analysis was used to check the specificity of PCR products. The used primers are listed in Table S1 (Additional file 1: Table S1).

### Western blot

Briefly, total proteins were extracted using RIPA (Radio-Immunoprecipitation Assay) lysis buffer containing 1 mM PMSF (Phenylmethanesulfonyl fluoride) (Beyotime, Beyotime Institute of Biotechnology, Shanghai, China) and 10% PhosSTOP phosphatase inhibitor Cocktail (Roche), and then resolved by electrophoresis through a 10% SDS-PAGE. The amount of separated proteins (20 μg per lane) was transferred onto 0.45 μM PVDF membranes (Millipore, USA) and incubated with primary antibody against α1A-adrenergic receptor (1:1000, Abcam), Collagen I (1:1000, Abcam), α-SMA (1:300, Abcam), α-AMPK (1:1000, CST, Cell Signal Technology), p- αAMPK (1:1000, CST), GSK-3β (1:1000, CST), p-GSK-3β (1:1000, Tyr 216) (Abcam), Cyclin D1 (1:10000, Abcam), c-Myc (1:10000, Abcam), Nanog (1:2000, CST), N-Cadherin(1:1000, Abcam), E-Cadherin(1:1000, Abcam), Vimentin (1:1000, Abcam), Snail (1:1000, Abcam), β-catenin (1:1000, CST), sFRP1 (1:1000, Abcam), Wnt 16B (1:1000, Bioss) or GAPDH (1:1000, Beyotime) and the corresponding HRP-conjugated secondary antibody (PeproTech). Protein band was developed using Ncm-ECL Ultra (New Cell & Molecular Biotech Co., Ltd., China).

### Cell proliferation assay

Cell proliferation was measured using the Cell Counting Kit-8 assay (CCK-8, Yeasen, Shanghai, China). Briefly, cells were seeded into 96-well plates (1 × 10^3^ cells /well) and cultured for the indicated time periods. Then, 10 μl of CCK-8 reagent was added into each well for 1 h at 37 °C. The absorbance was measured using a Multiskan spectrophotometer at a wavelength of 450 nm.

### Immunofluorescence assay

After fixed with 4% paraformaldehyde (Sangon, Shanghai, China), incubated with 0.3% Triton X-100 (Sangon, Shanghai, China) and blocked with 5% BSA (Sangon, Shanghai, China), the cells were incubated with primary antibody at 4 °C overnight, followed by incubation with the appropriate secondary antibody (Thermo Scientific). The nuclei were counterstained with 4, 6-diamidino-2-phenylindole (DAPI) (Yeasen, Shanghai, China). The intensity of fluorescence was detected using a confocal laser scanning microscopy (LSM510, Zeiss, Germany).

### Migration and invasion assays

For migration analyzed by wound-healing assay, the cell monolayer was mechanically disrupted to produce a linear wound using a sterile 200 μl pipette tip. The distance migrated by cells was measured using a microscope equipped with an ocular micrometer. For invasion assay, 1 × 10^5^ cells suspended in serum-free medium were seeded into the upper chamber coated with Matrigel (BD Biosciences, USA) in 24-well transwell plates (8-μm pore size, Corning, NY, USA), and 600 μl DMEM with 10% FBS was added into the lower chamber. After incubation for an indicated time points, the invading cells on the outer side of the upper chamber membrane were fixed with 4% paraformaldehyde, stained with crystal violet and counted under a light microscopy.

### Flow cytometric analysis

For analysis of apoptosis using Alexa Fluor 488 Annexin V Kit (Invitrogen), the cells (1 × 10^6^ cells/ml) were harvested, washed with PBS and centrifuged at 1000 rpm for 5 min. Then, the cell pellet was re-suspended in the annexin-binding buffer, incubated with annexin V and PI working solution for 15 min at room temperature. Cell apoptosis was determined using FACS caliber Flow cytometer (BD Biosciences, San Jose, CA, USA) and FlowJo software (Tree Star, San Carlos, CA).

### Microarray

The mRNA expression profiles were generated using Affymetrix GeneChip arrays (Affymetrix, Santa Clara, CA, US) according to the manufacturer’s instructions, as described previously [27].

### Enzyme-linked Immunosorbent assay (ELISA)

The sFRP1 level in conditioned media collected from cells treated with NE was measured by an ELISA kit (R&D Systems, Wiesbaden, Germany) following the manufacturer’s instructions. For NE, tissue was homogenized in 0.01 M HCl at 10% volume (ml) by tissue weight. NE in serum and tissue was quantified by ELISA (Elabscience, E-EL-0047c, China) according to the manufacturer’s protocol, as described previously [28].

### α-AMPK kinase blocking assay

For blocking experiments, LX2 cells were pre-incubated with Dorsomorphin (200 nM, 1 μM or 5 μM) (α-AMPK inhibitor, Selleck Chemicals, China) for 2 h prior to exposure to NE (10 μM) for another 4 h or 24 h.

### Inhibition of GSK-3β kinase and β-catenin

HCC cells were pre-treated with GSK-3β inhibitor CHIR-99021 (10 μM) or β-catenin inhibitor XAV-939 (10 μM) (Selleck Chemicals, China) for 2 h, followed by the treatment of NE (10 μM) for another 3 h or 24 h.

### Immunoprecipitation

To identify sFRP1-targeted molecules, co-immunoprecipitation was conducted as previously described. Briefly, HCC cells were lysed in immunoprecipitation buffer supplemented with a protease inhibitor for 2 h at 4 °C. The sample was centrifuged at 12,000 x g for 15 min, and the protein concentration was determined using the Bradford method (Beyotime, Beyotime Institute of Biotechnology, Shanghai, China). Precleared lysates with equivalent amounts of protein were incubated with a primary antibody overnight at 4 °C. Then, protein A- and G-Sepharose beads (Pierce Biotechnology, Rockford, IL, USA) were added to the immunoprecipitation (IP) mixture for 2 h. After that, the precleared lysate was incubated with a specific antibody coupled to protein A/G-agarose beads for 2 h at 4 °C. The precipitates were washed four times with immunoprecipitation buffer, resolved by 10% SDS-PAG, detected by western blot with specific antibody and visualized by enhanced chemiluminescence.

### Wnt16B promoter analysis

HCC cells were transfected (riboFECT™ CP Reagent, Guangzhou RiboBio Co., Ltd) with a plasmid containing a full-length sequence of Wnt16B promoter (2000 bp upstream of transcription start site) and subsequently assayed for luciferase reporter gene expression (Promega), as described previously [27].

### Immunohistochemistry

Immunohistochemistry was performed using the EnVision two-step Visualization System (GeneTech, Shanghai, China). Briefly, tumor specimens were removed, fixed in 10% neutral formalin and embedded with paraffin, and sliced into 5 mm thick sections. Sections were deparaffinized with xylene, followed by rehydration with a graduated series of ethanol, blocking endogenous peroxides with 3% H_2_O_2_, antigen-retrieval with microwave, blocking non-specific antibody binding. Slides were next incubated with primary antibodies against Cyclin D1 (1:50, Abcam), N-Cadherin(1:100, Abcam), E-Cadherin (1:100, Abcam), β-catenin (1:100, CST), sFRP1 (1:50, Abcam), overnight at 4 °C, followed by incubation with the secondary antibodies the next day, and visualized with 3,3-diaminobenzidine (DAB) as a chromogen. The slides were counterstained with hematoxylin.

### Animal experiments

All animal experiments were approved by the Ethical Committee on Animal Experiments of Animal Care Committee of Zhongshan Hospital of Fudan University, Shanghai, China and carried out according to the Shanghai Medical Experimental Animal Care Commission Guidelines. All Male BALB/c nude mice (4–6 weeks old and weighing 18–20 g) were purchased from SLAC Laboratory Animal Co., Ltd., Shanghai, China and housed in a pathogen-free condition, and all efforts were made to minimize animal suffering. In one experiment, mice were injected subcutaneously with a cell suspension containing 3× 10^7^ Huh7 cells with or without 1× 10^7^ LX-2 cells into the upper right flank portion of each mouse. After 5 days of tumor inoculation, mice were assigned to four groups: (a) Huh7 control group (*n* = 5); (b) Huh7 + daily stress group (*n* = 5); (c) Huh7/LX2 cells control group (*n* = 5); (d) Huh7/LX2 cells + daily stress group (*n* = 5). In the chronic stress, the mouse is restrained in a movement-restricted space using an acrylic cylindrical animal restrainer, which restricts the movement of the limbs with unlimited breathing. After restraint for 2 h, the mice were returned to their home cages and allowed access to food and water. The mice were subjected to daily restraint stress for 3 weeks as described previously [7]. In the second experiment, cell suspensions containing 3× 10^7^ Huh7 cells with 1× 10^7^ sFRP1 shRNA LX-2 (LX-2^shRNA sFRP1^) or scramble shRNA LX-2 (LX-2^shRNA NC^) cells were injected subcutaneously into mice (*n* = 5 for each group). After 1 week of inoculation, mice were subjected to chronic stress for 3 weeks as described above. The length and width of the tumors were measured twice per week, and the tumor volume was calculated according to the formula: (length × width^2^)/2. After 1 month, mice were sacrificed, and tumors, livers, and lungs were harvested, fixed with 10% formalin or frozen in liquid nitrogen for the following analyses.

### Human samples

Ethical approval from the Zhongshan Hospital of Fudan University (Shanghai, China) Research Ethics Committee and patient written informed consents were obtained from each patient. HCC and matched nontumor liver tissues were collected from 26 patients who underwent curative resection at the Liver Cancer Institute, Zhongshan Hospital of Fudan University (Shanghai, China) in 2015. The pathologic diagnosis of HCC was confirmed. Clinicopathological information was retrieved from the medical records.

### Statistical analysis

Data were expressed as means ± standard deviation (SD) from three independent experiments. All statistical analyses were performed using the GraphPad Prism Software (GraphPad Software, San Diego, CA). The unpaired Student’s *t* test, one-way analysis of variance (ANOVA) or Fisher’s exact test was conducted for comparison between groups wherever appropriate. All statistical tests were two-sided and a *P* < 0.05 was considered statistically significant.

## Results

### NE is capable of activating HSCs via the α1A-ADR

**Fig. 1:**
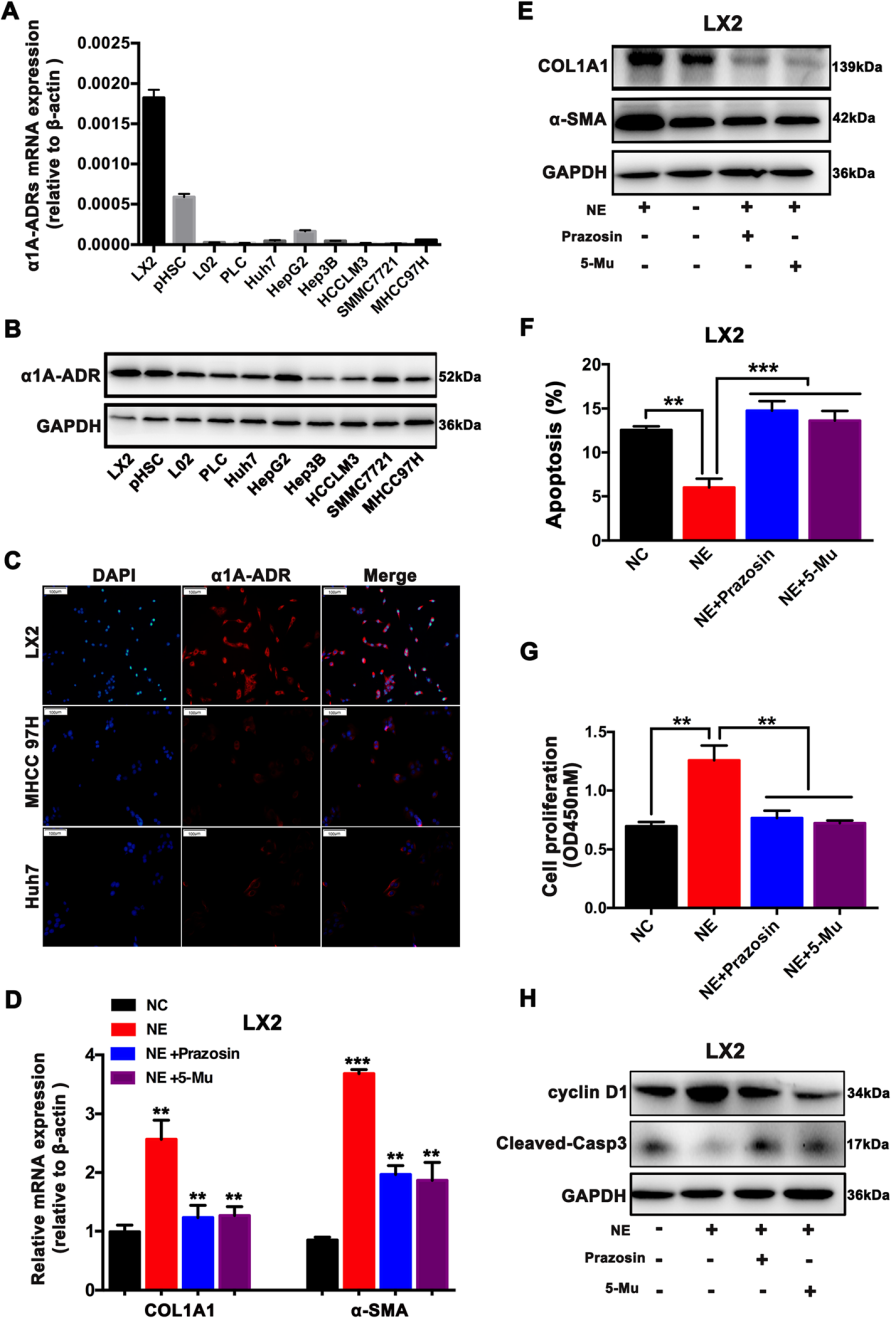
NE activates HSCs through α1A-ADR. **a**, **b** HSCs express a high level of a subtype of adrenergic receptors, α1A-ADR. The expression of α1A-ADR was significantly increased in HSCs (LX-2 and p-HSC) compared with HCC cells, as analyzed by qRT–PCR and western blot analysis. **c** Immunofluorescent staining of α1A-ADR in HSCs (LX-2 and p-HSC) (magnification, × 100). **d**, **e** Pretreated with α1-ADR antagonist prazosin (10 μM) or selective α1A-ADR antagonist 5-methylurapidi (5-Mu) (5 μM), LX-2 cells were exposed to 10 μM NE for 24 h. The mRNA and protein levels of αSMA and COL1A1 were determined by qRT-PCR analysis and western blot analysis. **f**, **g** Pretreated with α1-ADR antagonist prazosin or selective α1A-ADR antagonist 5-methylurapidi (5-Mu), LX-2 cells were treated with 10 μM NE for 24 h. Cell proliferation was measured by CCK-8 assay. Cell apoptosis was detected by flow cytometry using Annexin V/PI staining. **h** Protein expression levels of cyclinD1 and cleaved caspase 3 were determined by western blot analysis in LX-2 cells pretreated with prazosin or 5-Mu prior to 10 μM NE treatment. **P < 0.01, *** *P* < 0.001

The expression of adrenergic receptors (ADRs) was screened among HSCs (LX2 cells, p-HSC), liver cell line L02, and HCC cells (PLC/PRF/5, HepG2, Hep3B, Huh7 SMMC7721, MHCC97H, and HCCLM3). Compared with the varied expressions of α1B-, α1D-, β1-, β2- and β3-ADR in HSCs, liver cell line L02, and HCC cells (Additional file 2: Fig. S1), α1A-ADR mRNA expression was significantly upregulated in HSCs (Fig. 1a). Western blot analysis also confirmed the clear upregulation of α1A-ADR protein in HSCs (Fig. 1b). Positive immunofluorescence staining for α1A-ADR was observed in LX2 cells (Fig. 1c). NE treatment activated HSCs, as indicated by increased mRNA and protein expression of α-SMA and COL1A1 (primary signs of HSC activation), and enhanced cell proliferation (Additional file 3: Fig. S2). But, NE at the same concentrations as used for HSCs did not increase the proliferation of the HCC cells (Additional file 3: Fig. S2). More importantly, nonselective and selective α1-ADR antagonists (prazosin and 5-methylurapidi) efficiently suppressed NE-dependent HSC activation, as shown by reduced α-SMA and COL1A1 expression, reduced cell proliferation, and increased cell apoptosis in LX2 cells (Fig. 1d, e, f, g). In accordance with these changes, the α1-ADR antagonists significantly suppressed NE-induced cyclin D1 expression and remarkably increased the levels of cleaved caspase-3 (Fig. 1h). These results suggest that the stress hormone NE stimulates HSC activation through α1A-ADR signaling.

### The pro-tumoral effects of CM from NE-stimulated HSCs on the malignant characteristics of HCC cells is mediated by sFRP1

**Fig. 2:**
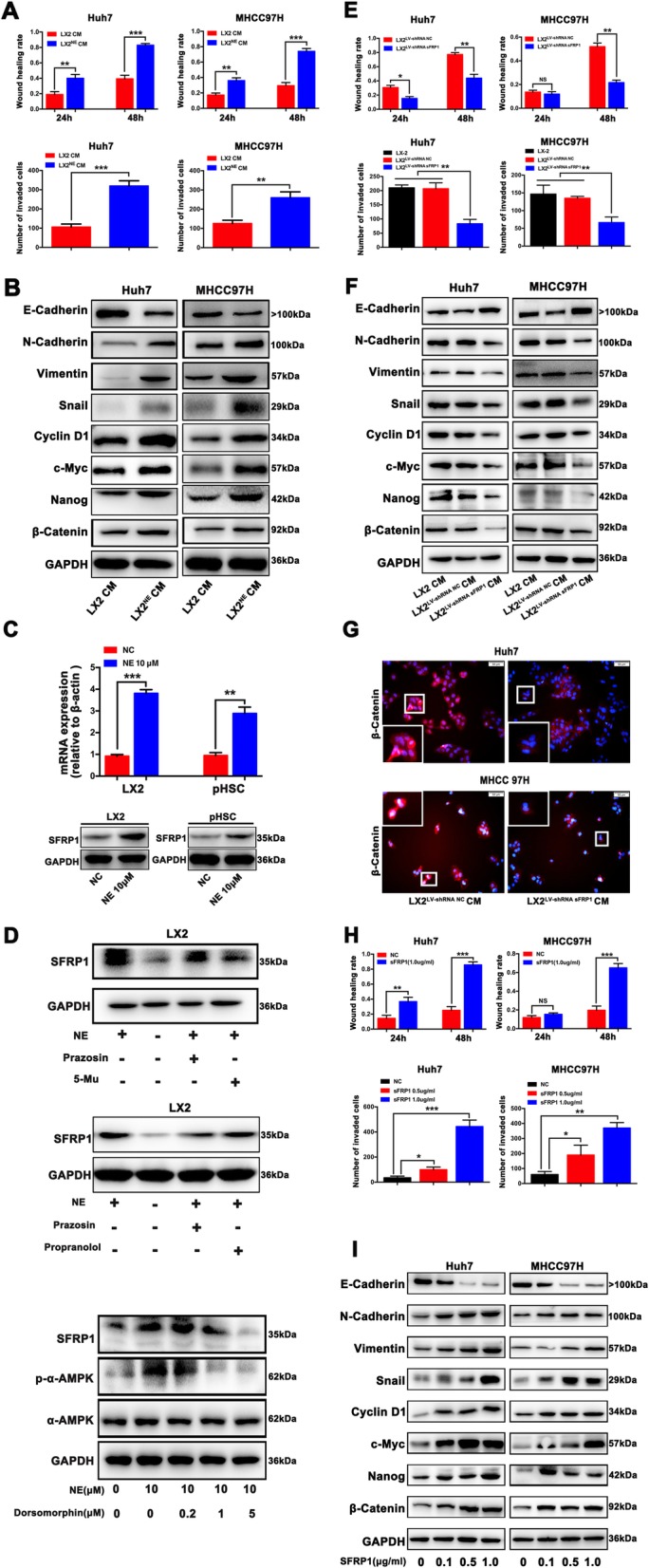
sFRP1 mediates the pro-tumoral effects of conditioned medium (CM) from NE-stimulated HSCs on malignant characteristics of HCC cells. **a** Compared with CM from NE-untreated LX2 cells, CM from NE-treated LX2 cells significantly enhanced the migration and invasion of HCC cells. **b** A decrease of E-cadherin expression and an increase of N-cadherin, vimentin, snail, c-Myc, cyclin D1, cancer stem cell markers of Nanog, and β-catenin were observed in HCC cells exposed to CM from NE-treated LX2. **c** NE (10 μM) significantly promoted the mRNA and protein expression of sFRP1 in HSCs (LX2 and p-HSC) compared with HCC cells, as analyzed by qRT-PCR. **d** Pretreated with prazosin (10 μM), 5-methylurapidi (5-Mu) (5 μM), propranolol (10 μM) or AMPK inhibitor dorsomorphin (0, 0.2, 1.0, 5.0 μM), LX-2 cells were treated with 10 μM NE. The expression of sFRP1 protein was detected by western blot analysis. **e** Compared with CM from NE-treated LX-2^shRNA NC^, CM from NE-treated LX-2^shRNA sFRP1^ showed a significant decrease of invasion and migration of HCC cells in vitro, as measured by wound-healing migration assay and Matrigel invasion assay. **f** The expression levels of EMT markers (E-cadherin, N-cadherin, vimentin, and snail), proliferation-related gene cyclin D1, cancer stem cell marker Nanog, and β-catenin were measured by western blot analyses in HCC cells exposed to CM from NE-treated LX-2^shRNA sFRP1^ versus NE-treated LX-2^shRNA NC^ cells. **g** Immunofluorescent staining showed that β-catenin nuclear translocation was significantly decreased when HCC cells were exposed to CM from NE-treated LX-2^shRNA sFRP1^ versus NE-treated LX-2^shRNA NC^. **h** Exogenous sFRP1 promoted the migration and invasion of HCC cells in vitro, as measured by wound-healing migration assay and Matrigel invasion assay. **i** A decrease in E-cadherin expression and an increase of N-cadherin, vimentin and snail expression, c-Myc, cyclin D1, Nanog and β-catenin were observed in HCC cells treated with exogenous sFRP1. *P < 0.05, **P < 0.01, *** *P* < 0.001

Compared with CM from NE-untreated LX2 cells (Control), CM from NE-treated LX2 cells significantly enhanced the migration and invasion of HCC cells in vitro (Fig. 2a; Additional file 4: Fig. S3). In parallel with the phenotypic changes, epithelial-mesenchymal transition (EMT) was indicated by a decrease of E-cadherin expression and increases of N-cadherin, vimentin and snail expressions. (Fig. 2b; Additional file 4: Fig. S3). In addition to EMT activation, β-catenin, proliferation-related genes c-Myc and cyclin D1, and cancer stem cell marker Nanog were markedly upregulated in HCC cells exposed to CM from NE-treated LX2 (Fig. 2b; Additional file 4: Fig. S3). To determine which secreted protein was responsible for these pro-tumoral effects in NE-treated LX2-cells CM, we performed gene expression profiles of NE-treated versus vehicle-treated LX-2 cells. A total of 31 differentially expressed genes that might act as paracrine signals from LX2 cells to HCC cells were identified (Additional file 5: Table S2). Of these, sFRP1, a regulator of the Wnt signaling [29–31], was selected based on the above finding that β-catenin was significantly increased after HCC cells exposed to CM from NE-treated LX2 (Fig. 2b). The levels of sFRP1 mRNA and protein expression were remarkably increased after HSCs (LX-2, p-HSC) were exposed to NE (Fig. 2c). By contrast, there was no significant difference of sFRP1 expression between NE-untreated and NE-treated HCC cells (Additional file 6: Fig. S4). In addition, compared with other sFRP family members (sFRP2, sFRP3, sFRP4, sFRP5), sFRP1 mRNA expression was substantially upregulated by NE in a dose-dependent manner in LX2 cells (Additional file 6: Fig. S4). Although sFRP2 was significantly upregulated in NE-treated LX2 cells (Additional file 6: Fig. S4), the extent of sFRP1 increase in NE-treated LX2 cells was significantly higher than that of sFRP2. Notably, nonselective and selective α1-ADR antagonists (prazosin and 5-methylurapidi) significantly decreased the expression of sFRP1 in NE-treated LX2 cells detected by ELISA and western blot, whereas β-ADR antagonist propranolol did not reduce the expression of sFRP1 in NE-treated LX2 cells (Fig. 2d; Additional file 6: Fig. S4), suggesting that NE stimulates sFRP1 secretion from HSCs through α1A-ADR signaling. Recent research suggests that α1-ADR stimulation enhances the activity of AMPK [32], Additionally, an AMPK inhibitor (dorsomorphin) significantly decreased the expression of sFRP1 in NE-treated LX2 cells (Fig. 2d). These results indicate that sFRP1 secretion form NE-treated HSCs is through the activation of α1A-ADR/AMPK signaling.

To further define the roles of sFRP1 in the NE-stimulated HSC CM promotion of malignant phenotypes of HCC cells, we transfected LX-2 cells with a sFRP1-shRNA lentivirus (LX-2^shRNA sFRP1^) or a scramble-shRNA lentivirus (negative control, LX-2^shRNA NC^). The efficiency of gene silencing was confirmed (Additional file 7: Fig. S5). CM from NE-treated LX-2^shRNA NC^ and LX-2^shRNA sFRP1^ cells were collected to culture with HCC cells (Huh7, MHCC 97H). Compared with CM from NE-treated LX-2^shRNA NC^, CM from NE-treated LX-2^shRNA sFRP1^ showed a significant decrease of invasion and migration of HCC cells in vitro (Fig. 2e; Additional file 7: Fig. S5). Consistently, EMT was significantly attenuated in HCC cells co-cultured with NE-treated LX-2^shRNA sFRP1^ versus NE-treated LX-2^shRNA NC^ CM, evidenced by the higher E-cadherin expression and lower N-cadherin, Vimentin, and Snail expression (Fig. 2f; Additional file 7: Fig. S5). In addition, β-catenin, the proliferation-related gene cyclin D1 and cancer stem cell marker Nanog were markedly reduced in HCC cells exposed to CM from NE-treated LX-2^shRNA sFRP1^ compared with those cultured with CM from NE-treated LX-2^shRNA NC^ (Fig. 2f; Additional file 7: Fig. S5). It has been reported that sFRP-1 regulates Wnt/β-catenin signaling [29, 33]. Western blot and immunofluorescence assays showed that β-catenin expression and its nuclear translocation were significantly decreased when HCC cells were exposed to CM from NE-treated LX-2^shRNA sFRP1^ (Fig. 2g). These results demonstrate that sFRP1 mediates the pro-tumoral effects of CM from NE-stimulated HSCs on malignant features of HCC cells.

On the contrary, the administration of exogenous sFRP1 promoted the migration and invasion of HCC cells in vitro (Fig. 2h; Additional file 7: Fig. S5). Concurrently, induction of EMT by sFRP1 in a dose-dependent manner in HCC cells was observed, as indicated by a decrease of E-cadherin expression and an increase of vimentin and snail expression (Fig. 2i). Further, c-Myc, cyclin D1, Nanog and β-catenin were significantly upregulated in HCC cells by sFRP1 treatment (Fig. 2i). In addition, CHIR 99021 and XAV939 influenced EMT, β-catenin activation induced by sFRP1 (Additional file 8: Fig. S6). These data suggest that exogenous sFRP1 mimicked the pro-tumoral effects of CM from NE-stimulated HSCs on malignant features of HCC cells and activation of β-catenin signaling.

Taken together, these data reveal that pro-tumoral effects of NE-stimulated HSCs on malignant characteristics of HCC cells are mediated by sFRP1, and activation of β-catenin contributes to these processes.

### sFRP1 augments an autocrine feedback loop of Wnt16B/β-catenin signaling in HCC cells

**Fig. 3:**
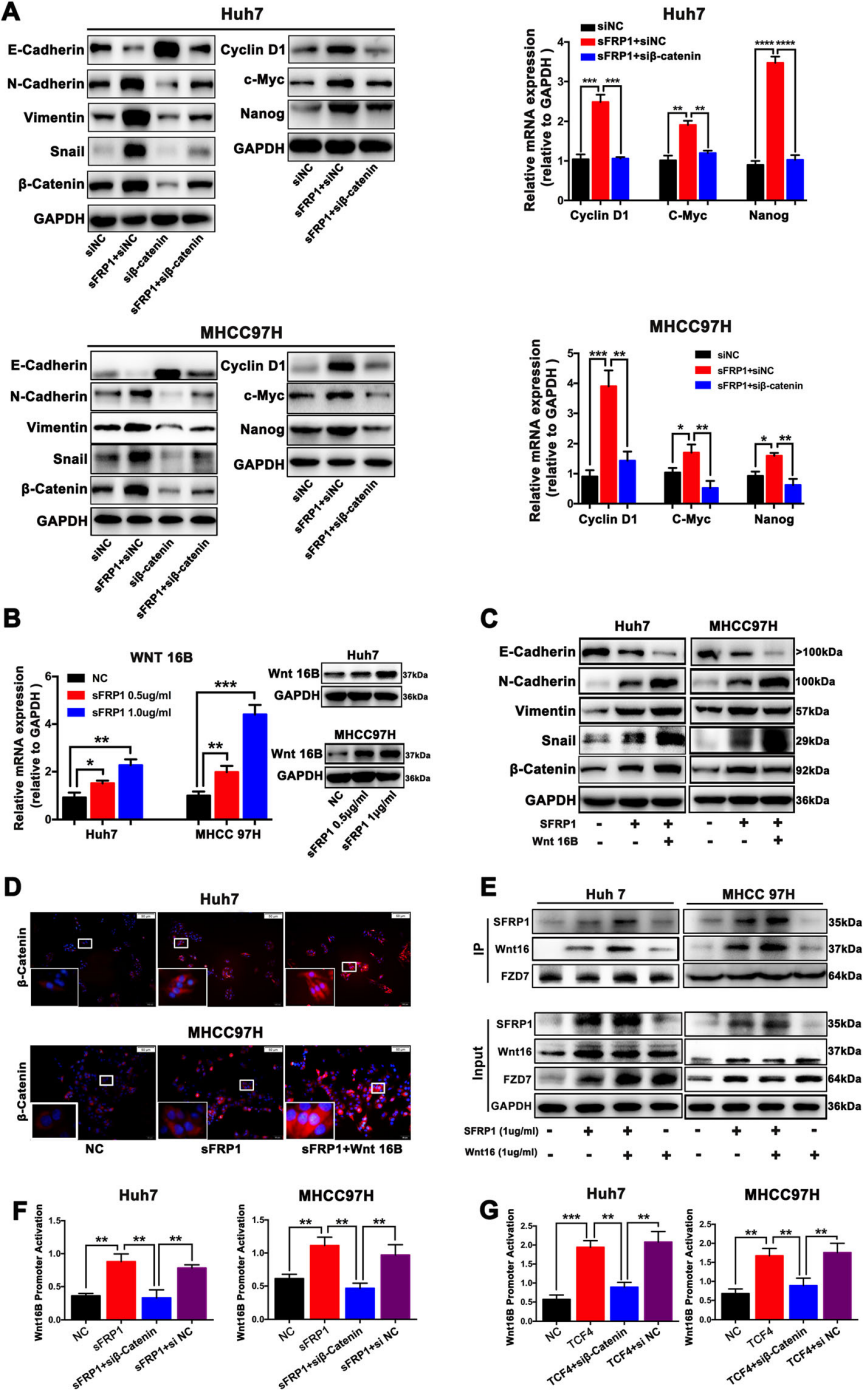
sFRP1 augments an autocrine feedback loop of Wnt16B/β-catenin signaling in HCC cells. **a** Downregulation of β-catenin by siRNA (siβ-catenin) significantly suppressed sFRP1-induced EMT, c-Myc, cyclin D1 and Nanog expression in HCC cells. **b** The mRNA and protein expression levels of Wnt16B were significantly increased in HCC cells treated with exogenous sFRP1 (0, 0.5,1.0 μg/mL), as detected by qRT-PCR and western blot analyses. **c** EMT-related markers (E-cadherin, N-cadherin, vimentin and snail) were assessed in HCC cells treated with sFRP1 alone or sFRP1 (1.0 μg/mL) and Wnt16B (0.5 μg/mL). **d** Nuclear β-catenin in HCC cells treated with sFRP1 (1.0 μg/mL) alone or sFRP1 and Wnt16B (0.5 μg/mL) were detected by immunofluorescence assays. **e** Wnt16B binding to FZD7 was significantly increased in the presence of sFRP1, as indicated by immunoprecipitation assay. It demonstrated that level of Wnt16B interacted with FZD7 was significantly upregulated in HCC cells in the presence of sFRP1, suggesting that sFRP1 strengthens FZD7 interaction with Wnt16B. **f** Downregulation of β-catenin by siRNA (siβ-catenin) significantly diminished sFRP1-induced Wnt16B promoter activity in HCC cells. **g** Over-expression of TCF4 enhanced Wnt16B promoter activity in HCC cells, which was inhibited by the downregulation of β-catenin (siβ-catenin). *P < 0.05, **P < 0.01, *** *P* < 0.001

As described previously, β-catenin activation may contribute to the pro-tumoral effects of sFRP1 on malignant phenotypes of HCC cells. Downregulation of β-catenin significantly suppressed the sFRP1-induced EMT, c-Myc, cyclin D1 and Nanog expression in HCC cells (Fig. 3a), suggesting that β-catenin mediates the effects of sFRP1 on HCC malignancy. To explore which Wnt family member is involved in sFRP1-activated signaling, we assessed the gene expression levels of 19 Wnt ligands in HCC cells treated with sFRP1. Wnt16B, one of the Wnt ligands, was chosen as the downstream effector based on the following reasons. As shown in Fig. S7 (Additional file 9: Fig. S7), Wnt1, Wnt3A, and Wnt16B were up-regulated in both sFRP1-treated HCC cells (MHCC97H and Huh7 cells). In addition, the relative base expression level of Wnt16B was higher than Wnt1 and Wnt3A. Also, Wnt16B mRNA expression in sFRP1-treated HCC cells was upregulated in a concentration-dependent manner. Wnt16B was remarkably elevated in sFRP1-treated HCC cells, which was confirmed by qRT-PCR and western blot (Fig. 3b). Interestingly, HCC cells treated with both sFRP1 and Wnt16B displayed more apparent EMT, evidenced by the lowest level of E-cadherin and the highest levels of N-cadherin, vimentin, and snail when compared those treated with sFRP1 or Wnt16B alone (Fig. 3c), suggesting a synergistic interaction between sFRP1 and Wnt16B. Immunofluorescence showed that sFRP1 treatment in the presence of Wnt16B further increased the accumulation of nuclear β-catenin in HCC cells (Fig. 3d). These data suggest that there exists a synergistic effect of sFRP1 and Wnt16B on augmenting β-catenin activity.

Next, we investigated how sFRP1 and Wnt16B reinforced β-catenin signaling. As shown in Fig. 3e, Wnt16B binding to FZD7 was significantly increased in the presence of sFRP1, suggesting that sFRP-1 enhances the interaction of Wnt16B with its receptor FZD7 for the induction of β-catenin activation. It has been reported that β-catenin induces Wnt-responsive genes in various types of cancer [34–36]. Wnt16B promoter activity in HCC cells was significantly elevated by sFRP1 (Fig. 3f). Downregulation of β-catenin significantly diminished the sFRP1-induced Wnt16B promoter activity (Fig. 3f), suggesting that sFRP-1 promotes transcriptional activation of Wnt16B through β-catenin. Because the β-catenin activity is largely mediated by the transcription factors TCF/LEF, we hypothesized that β-catenin/TCF4 binding to the Wnt16B promoter upregulates the transcriptional activation of Wnt16B based on the presence of predicted TCF4-binding motifs in the Wnt16B promoter. As shown in Fig. 3g, Wnt16B promoter activity in HCC cells was significantly elevated by TCF4. Downregulation of β-catenin significantly diminished the TCF4-induced Wnt16B promoter activity (Fig. 3g), suggesting that β-catenin promotes the transcriptional activation of Wnt16B through TCF4.

Taken together, these data indicate that sFRP1 augments an autocrine feedback loop of Wnt16B/β-catenin in HCC cells by increasing the interaction of Wnt16B with FZD7 and β-catenin/TCF4-mediated Wnt16B expression.

### sFRP1 in HSCs accelerates HCC progression following chronic stress in vivo

**Fig. 4:**
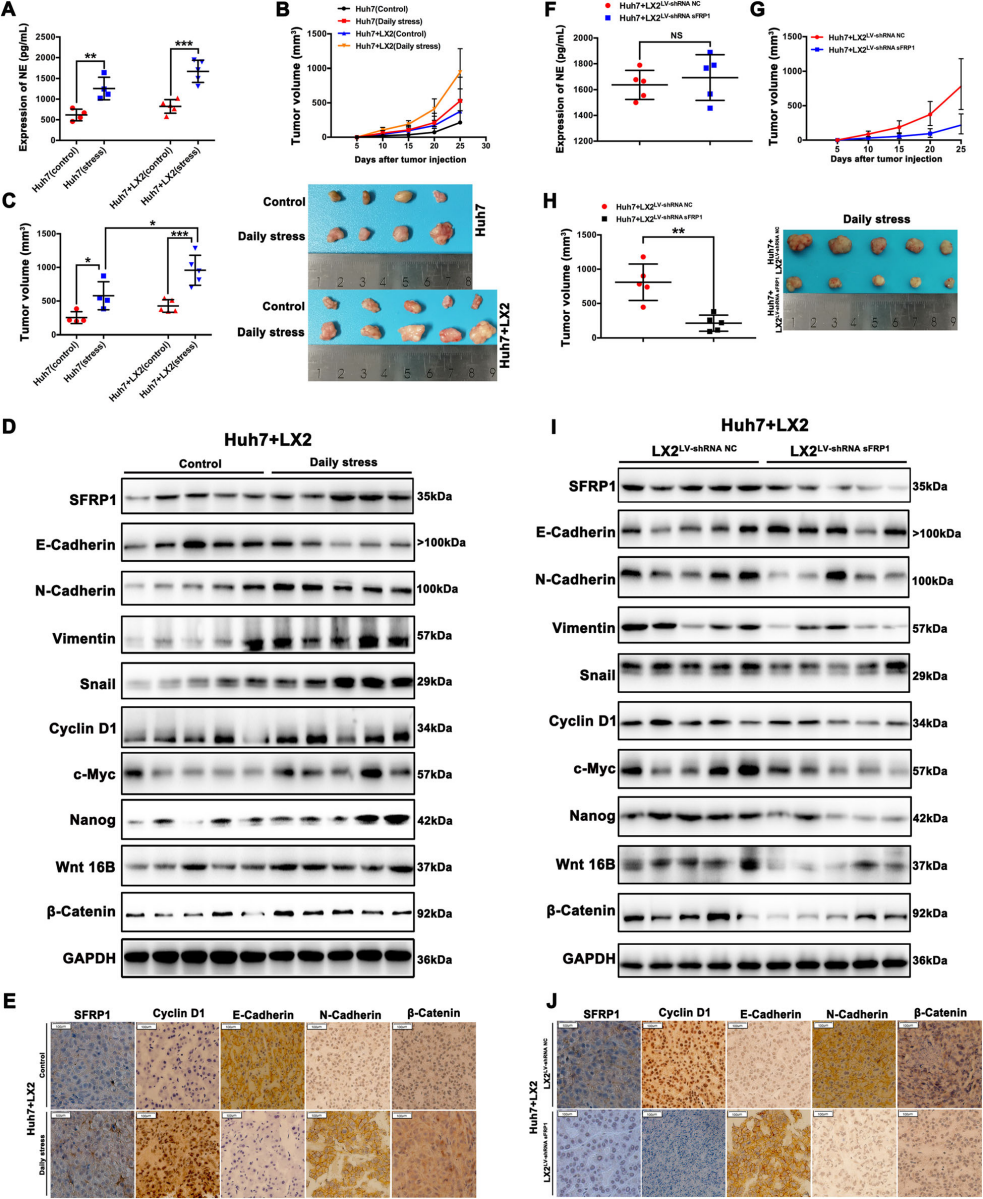
sFRP1 in HSCs accelerates HCC progression following chronic stress in vivo. **a** Serum NE levels in stressed or non-stressed mice were measured by ELISA. **b**, **c** Huh7 cells with or without LX2 cells were injected subcutaneously into the flanks of nude mice without or with chronic restraint stress. The tumor growth rates and tumor sizes are shown for mice in four groups: Huh7 (control), Huh7 (stress), Huh7 + LX2(control), and Huh7 + LX2(stress). **d**, **e** sFRP1, E-cadherin, N-cadherin, vimentin, snail, β-catenin c-Myc, cyclin D1, Nanog and Wnt16B expression levels in tumors from Huh7 + LX2(stress) versus Huh7 + LX2(control) groups were measured by western blot analysis, and sFRP1, cyclin D1, E-cadherin, N-cadherin, and β-catenin levels were validated using immunohistochemistry staining. **f** Huh7 + LX-2^shRNA sFRP1^ or Huh7 + LX-2^shRNA NC^ were injected subcutaneously into the flanks of nude mice with chronic restraint stress. Serum NE levels in mice subjected to chronic restraint stress were measured by ELISA. **g**, **h** The tumor growth rates and tumor sizes are shown for mice subjected to chronic restraint stress in two groups, Huh7 + LX-2 ^shRNA sFRP1^ versus Huh7 + LX-2^shRNA NC^. **i**, **j** sFRP1, E-cadherin, N-cadherin, vimentin, snail, β-catenin c-Myc, cyclin D1, Nanog and Wnt16B expression levels in tumors in stressed mice from Huh7 + LX-2 ^shRNA sFRP1^ versus Huh7 + LX-2^shRNA NC^ groups were measured by western blot analyses, and sFRP1, cyclin D1, E-cadherin, N-cadherin, and β-catenin were validated using immunohistochemistry staining. *P < 0.05, **P < 0.01, *** *P* < 0.001

We next investigated whether sFRP1 treatment promoted HCC progression in an in vivo model under chronic stress condition. Mice were subcutaneously injected with a cell suspension of Huh7 cells with or without LX2 cells and subjected to chronic restraint stress. The four groups of mice were all in a good general condition except two mice (one in Huh7 control group and one in Huh7 + daily stress group) died on the 15th day due to unknown reasons. The serum NE levels in the chronic restraint stress group (stress group) were significantly elevated compared with those in the control group (Fig. 4a). As shown in Fig. 4b, the growth rate of tumors was significantly accelerated in mice subjected to daily stress, particularly in the presence of LX2 cells. Similarly, regardless of tumors derived from Huh7 cells or Huh7 + LX2 cells, the size of tumors in the stress group was significantly increased when compared with the non-stress group (Fig. 4c), suggesting that stress promotes tumor progression. Notably, in mice subjected to stress condition, tumors derived from Huh7 + LX2 cells was significantly larger than tumors from Huh7 alone (Huh7 + LX2 + stress group versus Huh7 + stress group, *P* < 0.05; Fig. 4c), suggesting that the pro-tumor effect of stress-stimulated HSCs on HCC cells is more potent than the direct effect of stress itself on HCC cells. In Huh7/LX2 cells + daily stress group, sFRP1, N-cadherin, vimentin, snail, β-catenin c-Myc, cyclin D1, Nanog and Wnt16B expressions were significantly increased, whereas the expression of E-cadherin was markedly decreased (Fig. 4d). Consistent with the western blot results, sFRP1, N-cadherin, β-catenin, cyclin D1 were remarkably increased, whereas the expression of E-cadherin was significantly reduced in tumors in the Huh7/LX2 cells + daily stress group, as revealed by immunohistochemistry staining (Fig. 4e). To further define the role of sFRP-1 in stress-mediated HCC progression, Huh7 cell with LX-2 ^shRNA sFRP1^ cells or LX-2^shRNA NC^ cells were injected subcutaneously into stressed mice. Serum NE levels were similar between the two groups (Fig. 4f). After chronic restraint stress, the growth rates of tumors in the Huh7 cells+ LX-2^shRNA sFRP1^ group were remarkably slowed. Also, tumor sizes in the Huh7 cells+ LX-2^shRNA sFRP1^ group were significantly smaller than those in the Huh7+ LX-2^shRNA NC^ group (*P* < 0.05) (Fig. 4h). Additionally, sFRP1, N-cadherin, vimentin, snail, β-catenin c-Myc, cyclin D1, Nanog and Wnt16B were significantly reduced, whereas E-cadherin was increased in tumors of Huh7 cells+ LX-2^shRNA sFRP1^ versus Huh7+ LX-2^shRNA NC^ (Fig. 4i). According to immunohistochemical analyses, sFRP1, N-cadherin, β-catenin, cyclin D1 were significantly reduced, whereas E-cadherin was increased in tumors of Huh7 cells+ LX-2^shRNA sFRP1^ (Fig. 4j). These results demonstrate that chronic stress promotes sFRP1 increase in the presence of HSCs to enhance EMT, proliferation, cancer stem cell, and Wnt16B/β-catenin signaling in HCC, which is largely attenuated by knocking down sFRP1 in HSCs.

### sFRP1 expression in HCC tissues

**Fig. 5:**
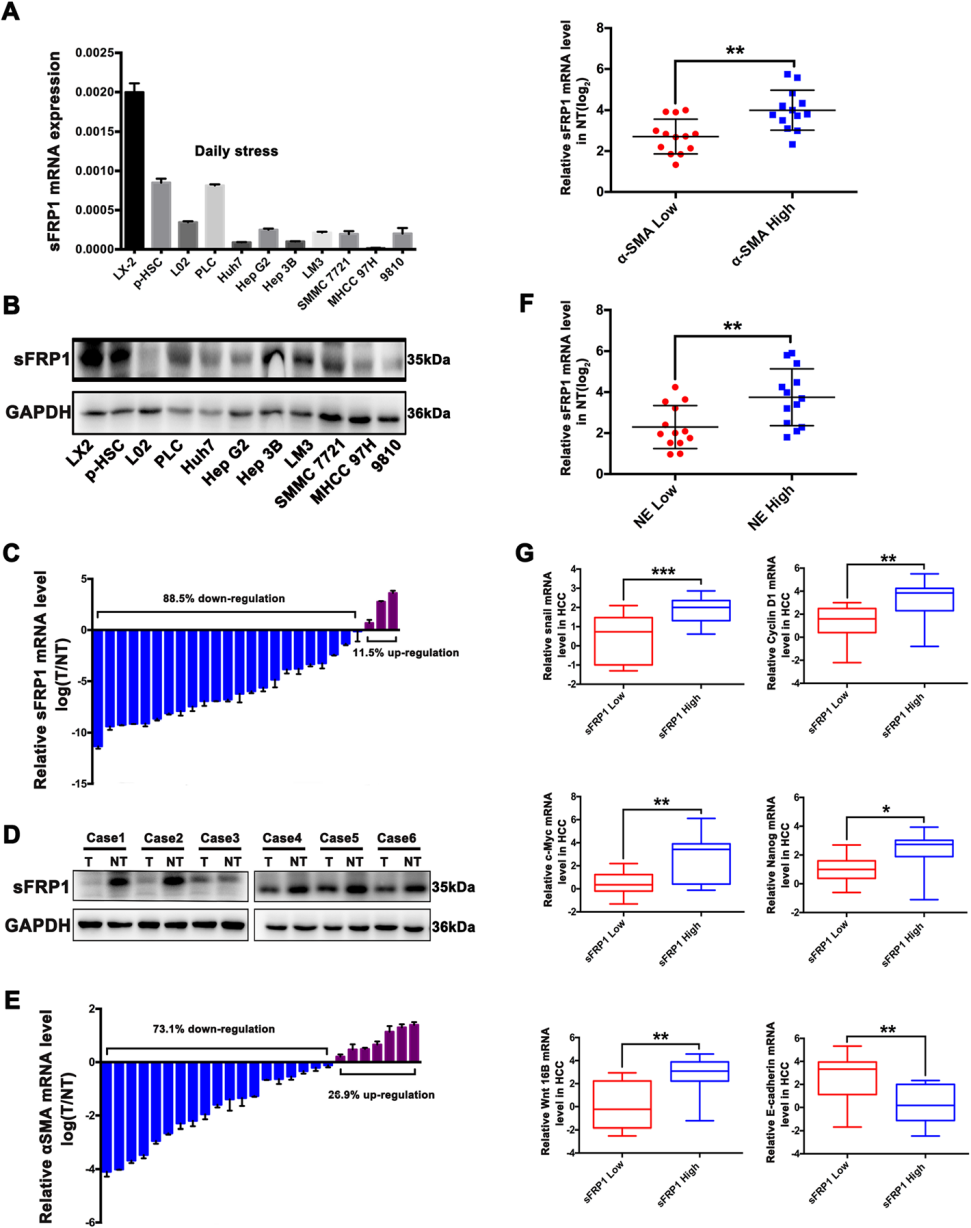
sFRP1 expression in HCC tissues. **a**, **b** The mRNA and protein expression levels of sFRP1 were measured in HSCs, L02 liver cells, and HCC cells. **c**, **d** The mRNA and protein expression levels of sFRP1 were upregulated in nontumoral liver tissues (NT) compared with HCC tissues (T), as measured by qRT-PCR and western blot analyses. **e** αSMA was highly expressed in NT. Using the median expression level of α-SMA in non-tumoral tissues as a threshold, the samples were classified into two groups. Compared with the low α-SMA group, sFRP1 was highly upregulated in the high α-SMA group. **f** Compared with the group with low NE levels in NT, sFRP1 was highly elevated in the group with high NE levels in NT. **g** Taking the median mRNA expression level of sFRP1 in the non-tumoral tissues as a threshold, we classified the cases into two groups: a low sFRP1 group and a high sFRP1 group. The snail, cyclin D1, c-Myc, Nanog, Wnt16B and E-cadherin in HCC tissues were assessed in the high versus low sFRP1 groups. *P < 0.05, **P < 0.01, *** *P* < 0.001

The baseline expression of sFRP1 in HSCs was significantly higher than that in the liver cell line L02 or in HCC cells (Fig. 5a, b), suggesting that the main source of sFRP1 is HSCs. To assess the clinical significance of sFRP1, we analyzed clinicopathological data in 26 patients with HCC and found that the expression of sFRP1 at both the mRNA and protein levels was remarkably higher in non-tumoral liver tissues than that in HCC tissues (Fig. 5c, d). Meanwhile, the expression of αSMA, a primary hallmark of HSC activation, was also highly expressed in non-tumoral liver tissues. Using the median expression level of α-SMA in non-tumoral tissues as a threshold, we classified the samples into two groups, a low HSC group, and a high HSC group. Compared with the low HSC group, sFRP1 was highly expressed in the high HSC group (*P* < 0.001, Fig. 5e), suggesting the increased sFRP1 in non-tumoral liver tissues may result from HSC production. Moreover, we grouped cases based on the median level of NE in non-tumoral tissues and found that high levels of NE in non-tumoral tissues were associated with a significant increase in sFRP1 expression (*P* < 0.05, Fig. 5f), suggesting that NE may increase sFRP1 in non-tumoral liver tissues. Considering the median mRNA expression level of sFRP1 in non-tumoral tissues as a threshold, we classified the cases into two groups, a low sFRP1 group, and a high sFRP1 group. In parallel, snail, cyclin D1, c-Myc, Nanog and Wnt16B levels in HCC tissues were significantly upregulated in the high sFRP1 group, whereas E-cadherin levels were clearly decreased (Fig. 5g). Additionally, a significant correlation was detected between the Vimentin/E-cadherin ratio and sFRP1 expression (*P* < 0.001) (Additional file 10: Fig. S8). These results suggest that NE-induced sFRP1 in non-tumoral liver tissues promotes EMT, proliferation, cancer stem cell, and Wnt16B/β-catenin signaling in HCC progression.

## Discussion

Associations between chronic stress and cancer progression have been studied [37, 38], and persistent SNS activation during chronic stress is known to contribute to the onset and progression of various diseases, including cancers [38]. In this study, we present a new evidence that chronic restraint stress promotes HCC progression through the activation of HSCs by the stress hormone NE. Specifically, NE-stimulated HSCs secrete sFRP1 to enhance HCC progression by augmenting an autocrine feedback loop of Wnt16B/β-catenin signaling. Our study suggests that sFRP1 may be a new potential therapeutic target for the treatment of chronic stress-induced HCC progression.

Several physiological problems, including fear, depression, and anxiety, are often seen in patients with cancer and can chronically activate stress pathways. Continuous exposure to stress enhances the progression of cancers, and this is partly mediated by NE and epinephrine released during increased sympathetic nervous activity as part of the body’s fight-or-flight stress response [39]. During stress, NE is locally secreted from nerve endings in various tissues and then into the blood, while epinephrine is secreted from the adrenal medulla [40–42]. In many types of tumors, NE, epinephrine, and activation of adrenergic receptors are involved in tumorigenesis, cancer survival, proliferation, angiogenesis, tumor progression, and metastasis through adrenergic receptor signaling-coupled intracellular molecular pathways [43, 44] or immunosuppression in the tumor microenvironment [45, 46]. Epidemiological data indicate the correlation between long-term survival of patients with cancer and β-blocker use (which antagonize NE/epinephrine receptors), suggesting a new class of potential cancer therapeutics [47]. Catecholamines, such as NE, affect not only cancer cells that express adrenergic receptors but also stromal cells within the tumor microenvironment. Different from previous studies [43], which focused on adrenergic receptor signaling in tumor cells, our study examined the effects of the stress hormone NE on HSCs, a type of stromal cells rich in the fibrotic tumor environment of HCC. Electron-microscopic studies have shown that HSCs, the major fibrogenic cells in the liver, are in contact with nerve fibers in the human liver [22]. In addition, several neuronal genes, including adrenergic receptors, are expressed in HSCs [48], and catecholamines are known to aggravate stress-induced liver diseases (e.g., cirrhosis) [49]. In this study, sFRP1 was chosen as the central molecule for the following reasons. CM from NE-treated HSCs promoted the malignant behaviors of HCC cells and increased β-catenin activity, suggesting that NE-stimulated HSCs may enhance HCC malignancies by a secreted/paracrine factor, which has the role in the alteration of Wnt/β-catenin pathway. After conducting differential gene expression analysis of NE-treated versus vehicle-treated LX-2 cells, we limited our search for candidate genes related to a secreted protein whose expression can modulate Wnt/β-catenin activity. Therefore, we selected sFRP1 as a downstream effector of NE-treated HSCs for its known roles in mediating Wnt/β-catenin signaling. To the best of our knowledge, this is the first study to report that NE up-regulates sFRP1 expression in HSCs through the activation of α1A-ADR signaling and that sFRP1 signaling from NE-stimulated HSCs promotes malignant characteristics of HCC cells (EMT, parameters of proliferation-related genes, and cancer stem cell markers). This study suggests sFRP1 as a potential target for interruption of chronic stress-promoted HCC progression.

Wnt signaling has been implicated in development, homeostasis, and disease [36] and sFRP1, a Wnt signaling regulator, is often considered a Wnt pathway inhibitor. Loss or downregulation of sFRP1 expression plays an important role in the development and progression of various cancer, including colon cancer [50], lung cancer [51], and HCC [52, 53]. However, sFRP1 also negatively regulates tumorigenesis and cancer progression [31, 54]. For example, during the development of prostate cancer, sFRP1 secreted from tumor stroma can provide a pro-proliferative signal to adjacent prostate epithelial cells [55]; in gastric cancer, crosstalk of sFRP1 with TGFβ signaling promotes cell proliferation, EMT and invasion. Furthermore, high levels of sFRP1 indicate aggressiveness in some subgroups of gastric cancer and poor survival of patients [56]. In this study, we presented the evidence that in the tumor microenvironment affected by NE, stromal HSCs within the tumor microenvironment of HCC acquire the capacity to secrete sFRP-1 and thereby promote phenotypic progression in HCC cells. Moreover, we found that sFRP1 from NE-stimulated HSCs augments an autocrine feedback loop of Wnt16B/β-catenin signaling in HCC cells by increasing the interaction of Wnt16B with the receptor FZD7 and enhancing Wnt16B expression, thereby inducing cell migration, invasion, EMT, and expression of proliferation-related markers and cancer stem cell markers to promote HCC progression. Regarding the mechanism, we found that following Wnt16B-mediated Wnt/β-catenin activation, sFRP1 further increased the nuclear translocation of β-catenin and subsequent enhancement of β-catenin/TCF4 signaling. Transcriptional activation of WNT16B was promoted by sFRP1-induced β-catenin/TCF4 signaling. Chronic restraint stress causes anxiety- and depression-like statuses in animal models. Our in vivo experiments further confirmed that sFRP1 in HSCs accelerated HCC progression in restraint-stressed mice. In accordance with the previous report that the density of α1A-ADR was increased in nontumoral liver tissues [57], we found that NE, α1A-ADR, α-SMA, COL1A1 (markers of HSCs activation) and sFRP1 were simultaneously over-expressed in nontumoral liver tissues, suggesting that HSCs in the peritumoral tissue may be the target of NE and the main source of sFRP1, whereas EMT, parameters of proliferation-related markers, and cancer stem cell markers, Wnt16B, and β-catenin were highly expressed in HCC tissues, suggesting that sFRP1 from HSCs in nontumoral liver tissues promotes HCC malignant characteristics. Due to a relatively small sample size of clinical HCC tissues (*n* = 26), the findings about the role of sFRP1 in HCC tissues need to be cautiously interpreted. Taken together, the in vitro and in vivo experiments and evaluation of clinical tumors demonstrate that NE-induced sFRP1 and subsequent initiation of Wnt16B/β-catenin signaling play significant roles in the effect of chronic stress on HCC progression, suggesting sFRP1 as a new target for blocking chronic stress-induced tumor progression.

## Conclusions

**Fig. 6:**
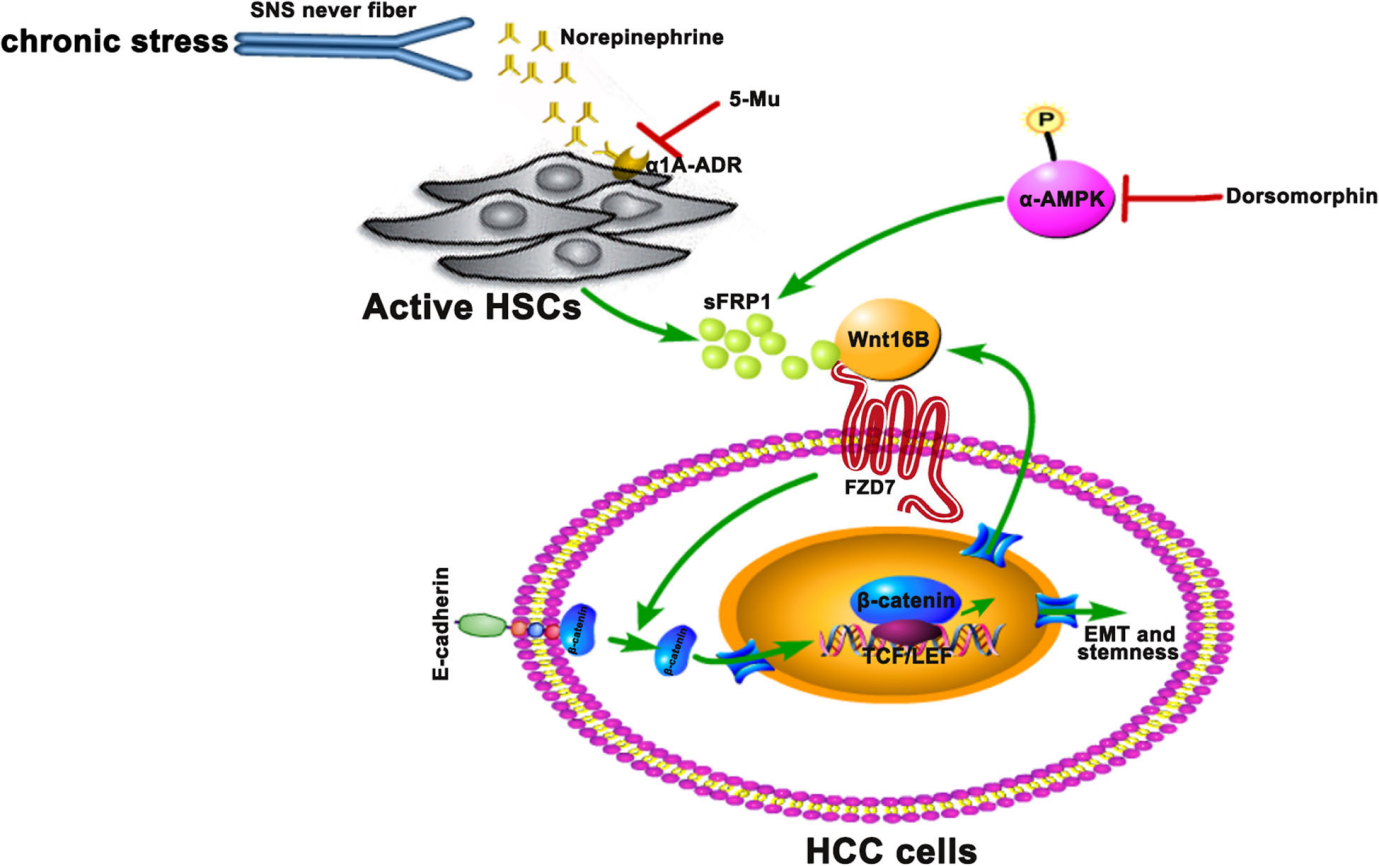
Schematic diagram of the proposed mechanism by which chronic stress promotes HCC progression

In conclusion (Fig. 6), we demonstrate that chronic stress promotes HCC progression, in which the stress hormone NE activates HSCs to secrete sFRP1, which cooperates with a Wnt16B/β-catenin positive feedback loop. Our findings have therapeutic implications for chronic stress-accelerated HCC progression.

## Supplementary information

**Additional file 1: Table S1.** Primers used for qRT-PCR

**Additional file 2: Figure S1.** The expression of adrenergic receptors (ADRs) in HSCs (LX2 cells, p-HSC), liver cell line L02, and HCC cells. (A) Varied expression of α1A-, α1B-, α1D-, β1-, β2-, and β3-ADR in HSCs, L02 liver cells, and HCC cells. (B) qRT-PCR analyses revealed that α1A-ADR and β2-ADR were relatively highly-expressed in LX-2 and p-HSC cells. (C) Immunofluorescent staining showed the expression of α1A-ADR in L02 and HCC cells (SMMC 7721 and HCC LM3) (magnification, × 100).

**Additional file 3: Figure S2.** NE treatment activated HSCs. (A, B) LX-2 cells were treated with 0.1, 1, 5, and 10 μM NE for 24 h. The mRNA and protein levels of αSMA and COL1A1 were determined by qRT-PCR and western blot analyses. (C) NE treatment enhanced cell proliferation. (D) NE treatment did not increase the proliferation of HCC cells.

**Additional file 4: Figure S3.** CM from NE-treated LX2 cells promoted the malignant phenotypes of HCC cells. (A, B) Compared with CM from NE-untreated LX2 cells, CM from NE-treated LX2 cells significantly enhanced the migration and invasion in Huh7 and MHCC 97H cells. (C, D) The expression of EMT markers (E-cadherin, N-cadherin, vimentin, snail, slug, ZEB1, and Twist), a stemness marker Nanog, and target genes of Wnt/β-catenin signaling (Axin2, c-Myc, CCND1, CD44, and LEF1) were measured by qRT-PCR in Huh7 and MHCC 97H cells co-cultured with CM from LX-2 cells versus CM from NE-treated LX-2 cells.

**Additional file 5: Table S2.** A total of 31 differentially expressed genes were identified in NE-treated versus vehicle-treated LX-2 cells.

**Additional file 6: Figure S4.** sFRP1 expression in NE- treated HCC cells or LX2 cells. (A) There was no significant difference of sFRP1 expression between NE-untreated and NE- treated HCC cells. (B) Compared with other sFRP family members (sFRP2, sFRP3, sFRP4, and sFRP5), sFRP1 mRNA expression was substantially upregulated by NE in a dose-dependent manner in LX2 cells. (C) Pretreated with prazosin (10 μM) or propranolol (10 μM), LX-2 cells were treated with 10 μM NE. The expression of sFRP1 was detected by ELISA. (D) Pretreated with prazosin (10 μM) or 5-methylurapidi (5-Mu) (5 μM), LX-2 cells were treated with 10 μM NE. The protein expression of sFRP1 was detected by ELISA.

**Additional file 7: Figure S5.** CM from NE-treated LX-2^shRNA sFRP1^ cells showed an attenuated promotion of malignant phenotypes of HCC cells in vitro**. (A)** LX-2 cells transfected with a sFRP1-shRNA lentivirus or a scramble-shRNA lentivirus. The efficiency of sFRP1 knockdown was examined in LX-2^shRNA sFRP1^ and LX-2^shRNA NC^ cells. (B, C) Compared with CM from NE-treated LX-2^shRNA NC^, CM from NE-treated LX-2^shRNA sFRP1^ showed a significant decrease of invasion and migration of HCC cells in vitro, as measured by wound-healing migration assay and Matrigel invasion assay. (D, E) qRT-PCR analyses were used to detect the expression of EMT markers (E-cadherin, N-cadherin, vimentin, snail, slug, ZEB1, and Twist), stemness marker Nanog and target genes of Wnt/β-catenin signaling (Axin2, c-Myc, CCND1, CD44, and LEF1) in Huh7 and MHCC 97H cells exposed to CM from LX-2^shRNA sFRP1^ versus LX-2^shRNA NC^ versus LX2. (F, G) Exogenous sFRP1 promoted the migration and invasion of HCC cells in vitro, as measured by wound-healing migration assay and Matrigel invasion assay.

**Additional file 8: Figure S6.** CHIR 99021 and XAV939 influenced EMT and β-catenin activation induced by sFRP1.

**Additional file 9: Figure S7.** Expression of Wnt family members in HCC cells exposed to sFRP1. (A, B) qRT-PCR analyses showed the expression levels of 19 Wnt family members in Huh7 cells exposure to 0.1, 0.5, or 1 μg/mL sFRP1 for 24 h. Fold changes represent the extent of relative mRNA change. (C) Wnt1, Wnt3A and Wnt16B were up-regulated in both sFRP1-treated HCC cells (MHCC97H and Huh7 cells). (D, E) There was a significant increase of sFRP1 in LX-2 cells treated with NE (0, 5, and 10 μM) whereas no significant change of Wnt16B was observed, as detected by qRT-PCR and western blot.

**Additional file 10: Figure S8.** sFRP1 expression in non-tumoral tissues associated with EMT in HCC. Taken the median mRNA expression level of sFRP1 in non-tumoral tissues as a threshold, we classified the cases into two groups, a low sFRP1 group and a high sFRP1 group. Using the ratio of the mRNA expression of Vimentin and E-cadherin (Vimentin /E-cadherin) as an indicator of EMT, Vimentin /E-cadherin ratio in HCC tissues was significantly upregulated in the high sFRP1 group.

## Data Availability

The datasets used and/or analyzed during the current study are available from the corresponding author on reasonable request.

## Abbreviations

EMT: Epithelial-mesenchymal transition
HCC: Hepatocellular carcinoma
HSCs: Hepatic stellate cells
CM: Conditioned medium
SNS: Sympathetic nervous system
α-ARs: alpha-adrenergic receptors
β-ARs: beta-adrenergic receptors
COL1A1: collagen I
α-SMA: alpha-smooth muscle actin
sFRP1: secreted frizzled-related protein 1
ADRA1A: α1A-adrenergic receptor
NE: Norepinephrine
E: Epinephrine
PRZ: Prazosin
5-Mu: 5-methylurapidil
