# Supplementary material for: Norepinephrine-stimulated HSCs secrete sFRP1 to promote HCC progression following chronic stress via augmentation of a Wnt16B/β-catenin positive feedback loop
Source: J Exp Clin Cancer Res. 2020 Apr 15;39:64. doi: 10.1186/s13046-020-01568-0 (PMC7158101; doi:10.1186/s13046-020-01568-0)
Supplement: Supplementary file 1 — Additional file 1: Table S1. Primers used for qRT-PCR [file 13046_2020_1568_MOESM1_ESM.docx]

**Table S1. Primers used for qRT-PCR**

| **Gene symbol** | **Primers (sequence 5’-3’)** |
| --- | --- |
| ADRA1A | Forward：GCAAGTGACGCTCCGCATCC |
| ADRA1B | Reverse：CGAAGCAGCCGACCACGATG  Forward：CTTCTTCATCGCTCTACCGCTTGG |
| ADRA1D  ADRB1  ADRB2  ADRB3  COL1A1  α-SMA  E-cadherin  N-cadherin  Vimentin  Nanog | Reverse：GTCTTCGTGCTCTGCTGGTTCC  Forward：GTCTTCGTGCTCTGCTGGTTCC  Reverse：GAAGTAGCCGAGCCAGAAGATGAC  Forward：CGCCTCGTCCGTAGTCTCCTTC  Reverse：GTCGATCTTCTTCACCTGCTTCTGG  Forward：CTCCAGCAACGGCAACACAGG  Reverse：ATCAATGTTATCGCTAGGCACAGTACC  Forward：GCTGTGCCTTCGCCTCCAAC  Reverse：GTAGACGAAGAGCATCACGAGAAGAG  Forward：GCGAGAGCATGACCGATGGATTC  Reverse：GCCTTCTTGAGGTTGCCAGTCTG  Forward：CTCTGGACGCACAACTGGCATC  Reverse：CACGCTCAGCAGTAGTAACGAAGG  Forward：GCTCTTCCAGGAACCTCTGTGATG  Reverse：AAGCGATGGCGGCATTGTAGG  Forward：AAGGTGGATGAAGATGGCATGGTG  Reverse：TGCTGACTCCTTCACTGACTCCTC  Forward：TTGCCGTTGAAGCTGCTAACTACC  Reverse：AATCCTGCTCTCCTCGCCTTCC  Forward：AGGATAGGTTTCAGAGGCAAA  Reverse：CCGTTGCTAGTCTTCAACCATTG |
| Sanil  Slug  ZEB1  Twist  Axin 2  c-Myc  LEF1  CCND1  SFRP1  SFRP2  SFRP3  SFRP4  SFRP5    WNT1  WNT2  WNT2B  WNT3  WNT3A  WNT4  WNT5A  WNT5B  WNT6  WNT7A  WNT7B  WNT8A  WNT8B  WNT9A  WNT9B  WNT10A  WNT10B  WNT11  WNT16B  GAPDH | Forward：GGCTCCTTCGTCCTTCTCCTCTAC  Reverse：CCAGGCTGAGGTATTCCTTGTTGC  Forward：CCATGCCTGTCATACCACAACCAG  Reverse：GGAGGAGGTGTCAGATGGAGGAG  Forward：GAGGAGGAGGAGGAGGAAGAAGTG  Reverse：CACTTGCTCACTACTCTCGCCTAC  Forward：GTCCGCAGTCTTACGAGGAG  Reverse：CCAGCTTGAGGGTCTGAATC  Forward：CACCACCACCACCACCATTCG  Reverse：ACATGCTTCGTCGTCTGCTTGG  Forward：CGAGGAGAATGTCAAGAGGCGAAC  Reverse： GCTTGGACGGACAGGATGTATGC  Forward：ACGTGAAGCCTCAGCATGAACAG  Reverse：ATGCCACCTTCTGCCAAGAATCTG  Forward：AACTACCTGGACCGCTTCCT  Reverse：CCACTTGAGCTTGTTCACCA  Forward：CGAGCCGGTCATGCAGTTCTTC  Reverse：TCGCTGGCACAGAGATGTTCAATG  Forward：GCCACGGCATCGAATACCAGAAC  Reverse：GCGAAGAGCGAGCACAGGAAC  Forward：CGATCTGCTCTTCTTCCTCTGTGC  Reverse：CACGAGTGGCGGTACTTGATGAG  Forward：CACCACAGCACGCAGGAGAAC  Reverse：CGTACATGGCACAGAGGAAGAAGC  Forward：GCCGCAGTGCCTTGACATCC  Reverse：CTGCTCGCCTGCTGCTTCAC  Forward：CTCCACGAACCTGCTTACAGACTC  Reverse：GACAGTTCCAGCGGCGATTCC  Forward：TCTCGGTGGAATCTGGCTCTGG  Reverse：TGGCTAATGGCACGCATCACATC  Forward：CCACTGGTGCTGTGCTGTACG  Reverse：GTCCAGCCACTCTGCCTTCTTG  Forward：CGGCTGTGACTCGCATCATAAGG  Reverse：GCCTCGTTGTTGTGCTTGTTCATG  Forward：CTGGTGGTCGCTGGCTGTTG  Reverse：GGCATGATCTCCACGTAGTTCCTG  Forward：TCCTCGTCTTCGCCGTCTTCTC  Reverse：CCATGACTTCCAGGTTCCGCTTG  Forward：GCTGGCAGACTTCCGCAAGG  Reverse：GAAGCGGCTGTTGACCTGTACC  Forward：GCCAACTCCTGGTGGTCATTAGC  Reverse：ACTGGTGCTGGCATTCCTTGATG  Forward：TCCTCTACGCCGCCGATTCG  Reverse：CTACGCAGCACCAGTGGAAGC  Forward：GGCTACGTGCTCAAGGACAAGTAC  Reverse：GCGGTACGACAGTGGCTTCTTG  Forward：GTGCCAGTACCAGTTCCGCTTC  Reverse：GCCGCAGTTGCTCAGGTTCC  Forward：TGCCTACAGAACAGCCACAACAC  Reverse：ACATGCCTACACTGGTCACACTTG  Forward：CAGCCATGAACCTGCACAACAAC  Reverse：GTGCTGCGTGGTACTTCTCCTTC  Forward：TGCCTTCCTCTATGCCATCTCCTC  Reverse：TCCTTGACGAACTTGCTGCTGTAC  Forward：GGCATCAAGGCTGTGAAGAGTGG  Reverse：CCTCATTGGTGGCACTGGACAC  Forward：GCGAGAATGAGGCTTCACAACAAC  Reverse：CACCTGCCAGCACGTCTTGAG  Forward：TCCTGACTTCTGTGAGCGAGACC  Reverse：CATAGCAGCACCAGTGGAAGCG  Forward：GATGTGGCTGCTGACCTCAAGAC  Reverse：AGACGAGTTCCGAGTCCTTCACAG  Forward：AGTGCAGGCAACATGACAGAGTG  Reverse：TCTGCTGAACCACATGCCATACTG  Forward：TGCCACTCAGAAGACTGTGG  Reverse：TTCAGCTCTGGGATGACCTT |
