# Supplementary figures and images for: Norepinephrine-stimulated HSCs secrete sFRP1 to promote HCC progression following chronic stress via augmentation of a Wnt16B/β-catenin positive feedback loop
Source: J Exp Clin Cancer Res. 2020 Apr 15;39:64. doi: 10.1186/s13046-020-01568-0 (PMC7158101; doi:10.1186/s13046-020-01568-0)

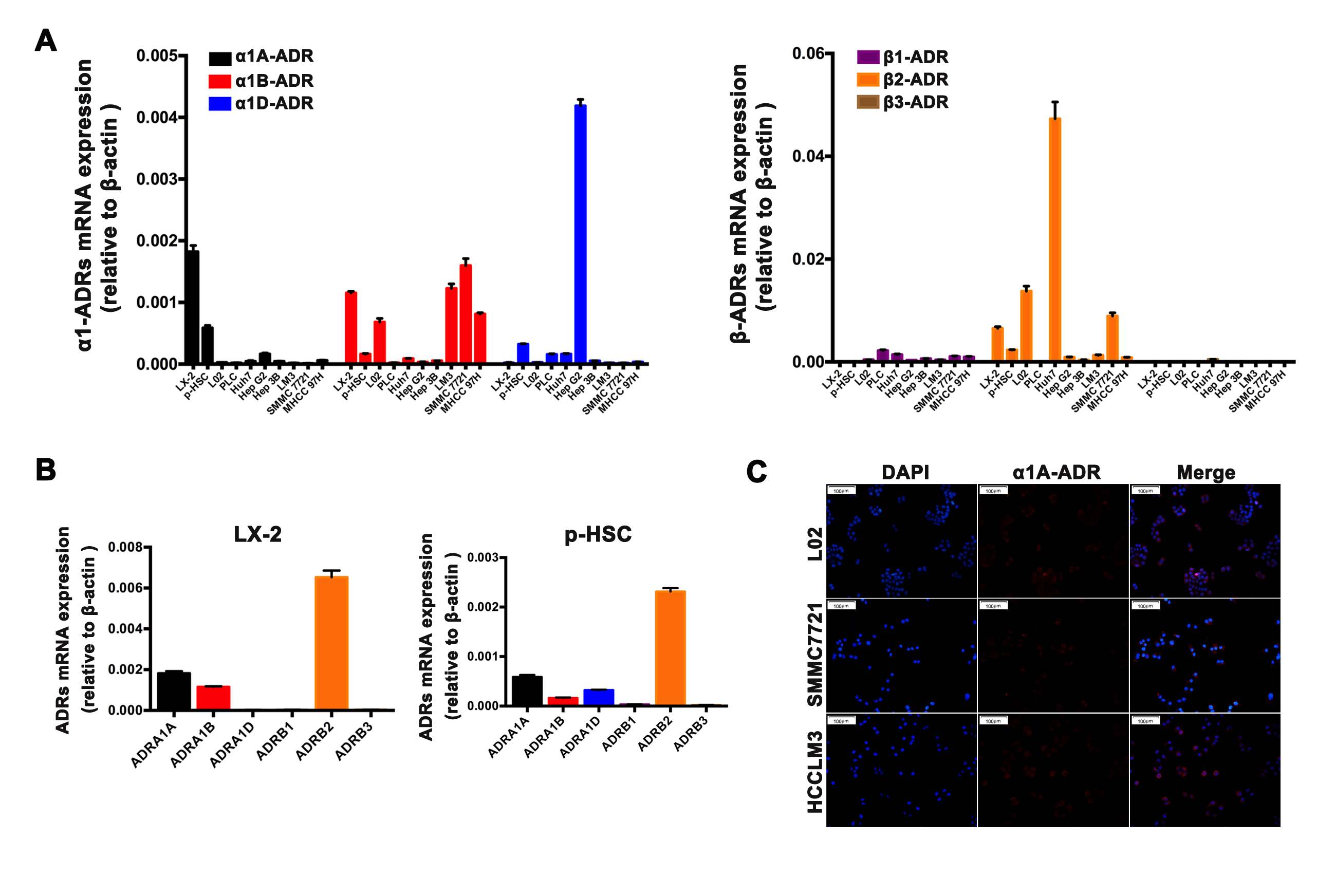

Supplement: Supplementary file 2 — Additional file 2: Figure S1. The expression of adrenergic receptors (ADRs) in HSCs (LX2 cells, p-HSC), liver cell line L02, and HCC cells. (A) Varied expression of α1A-, α1B-, α1D-, β1-, β2-, and β3-ADR in HSCs, L02 liver cells, and HCC cells. (B) qRT-PCR analyses revealed that α1A-ADR and β2-ADR were relatively highly-expressed in LX-2 and p-HSC cells. (C) Immunofluorescent staining showed the expression of α1A-ADR in L02 and HCC cells (SMMC 7721 and HCC LM3) (magnification, × 100). [file 13046_2020_1568_MOESM2_ESM.tif]

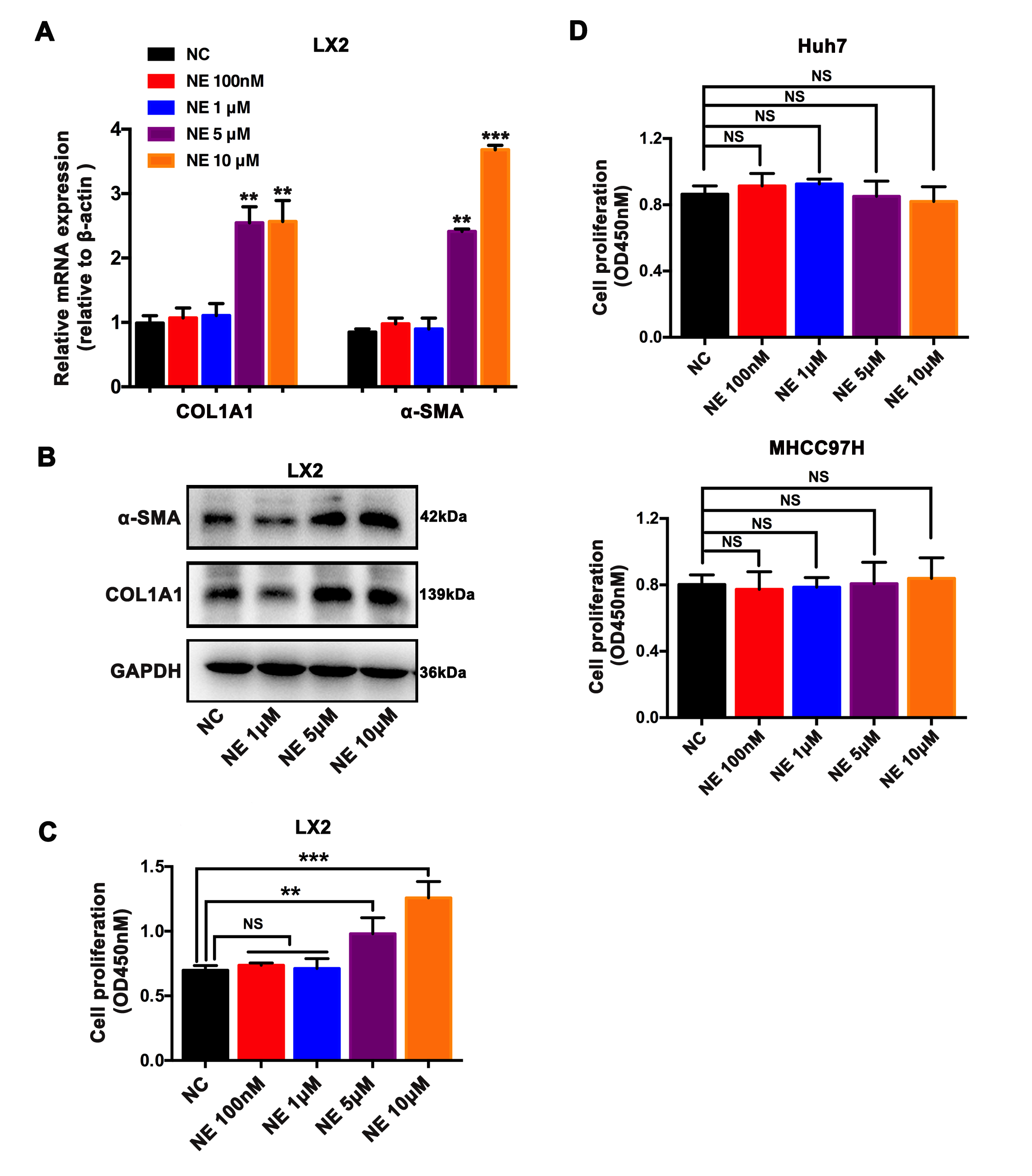

Supplement: Supplementary file 3 — Additional file 3: Figure S2. NE treatment activated HSCs. (A, B) LX-2 cells were treated with 0.1, 1, 5, and 10 μM NE for 24 h. The mRNA and protein levels of αSMA and COL1A1 were determined by qRT-PCR and western blot analyses. (C) NE treatment enhanced cell proliferation. (D) NE treatment did not increase the proliferation of HCC cells. [file 13046_2020_1568_MOESM3_ESM.tif]

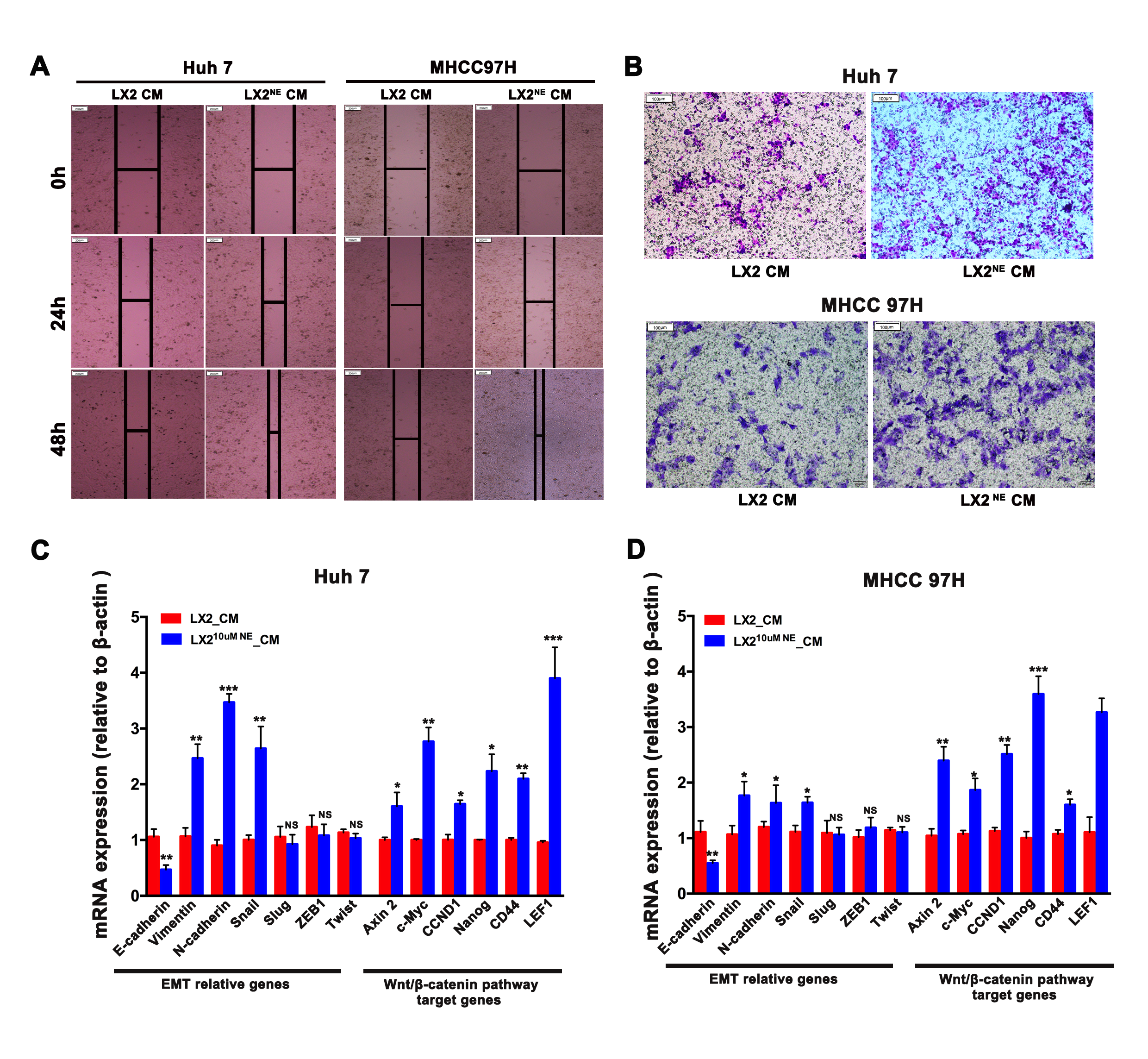

Supplement: Supplementary file 4 — Additional file 4: Figure S3. CM from NE-treated LX2 cells promoted the malignant phenotypes of HCC cells. (A, B) Compared with CM from NE-untreated LX2 cells, CM from NE-treated LX2 cells significantly enhanced the migration and invasion in Huh7 and MHCC 97H cells. (C, D) The expression of EMT markers (E-cadherin, N-cadherin, vimentin, snail, slug, ZEB1, and Twist), a stemness marker Nanog, and target genes of Wnt/β-catenin signaling (Axin2, c-Myc, CCND1, CD44, and LEF1) were measured by qRT-PCR in Huh7 and MHCC 97H cells co-cultured with CM from LX-2 cells versus CM from NE-treated LX-2 cells. [file 13046_2020_1568_MOESM4_ESM.tif]

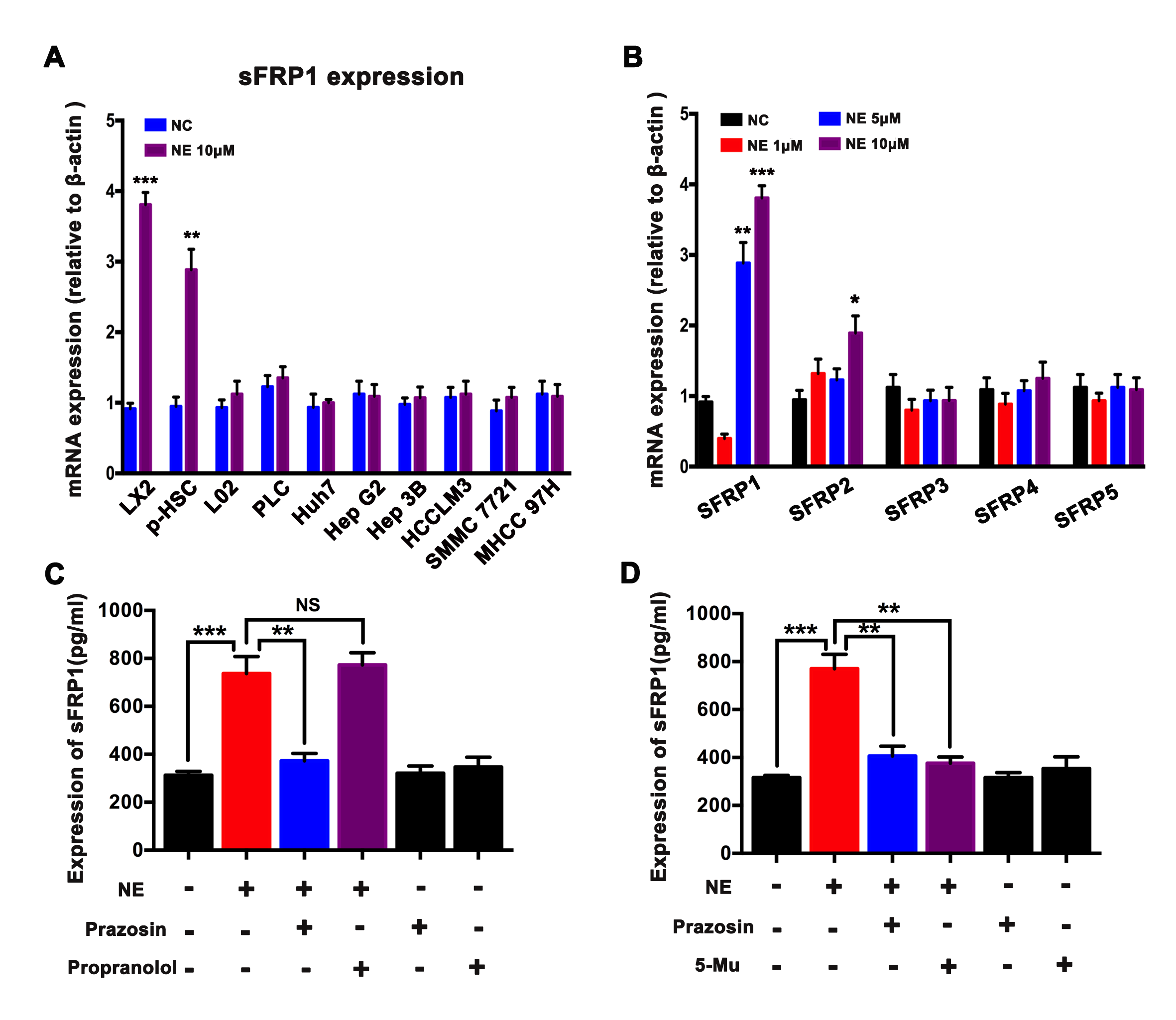

Supplement: Supplementary file 6 — Additional file 6: Figure S4. sFRP1 expression in NE- treated HCC cells or LX2 cells. (A) There was no significant difference of sFRP1 expression between NE-untreated and NE- treated HCC cells. (B) Compared with other sFRP family members (sFRP2, sFRP3, sFRP4, and sFRP5), sFRP1 mRNA expression was substantially upregulated by NE in a dose-dependent manner in LX2 cells. (C) Pretreated with prazosin (10 μM) or propranolol (10 μM), LX-2 cells were treated with 10 μM NE. The expression of sFRP1 was detected by ELISA. (D) Pretreated with prazosin (10 μM) or 5-methylurapidi (5-Mu) (5 μM), LX-2 cells were treated with 10 μM NE. The protein expression of sFRP1 was detected by ELISA. [file 13046_2020_1568_MOESM6_ESM.tif]

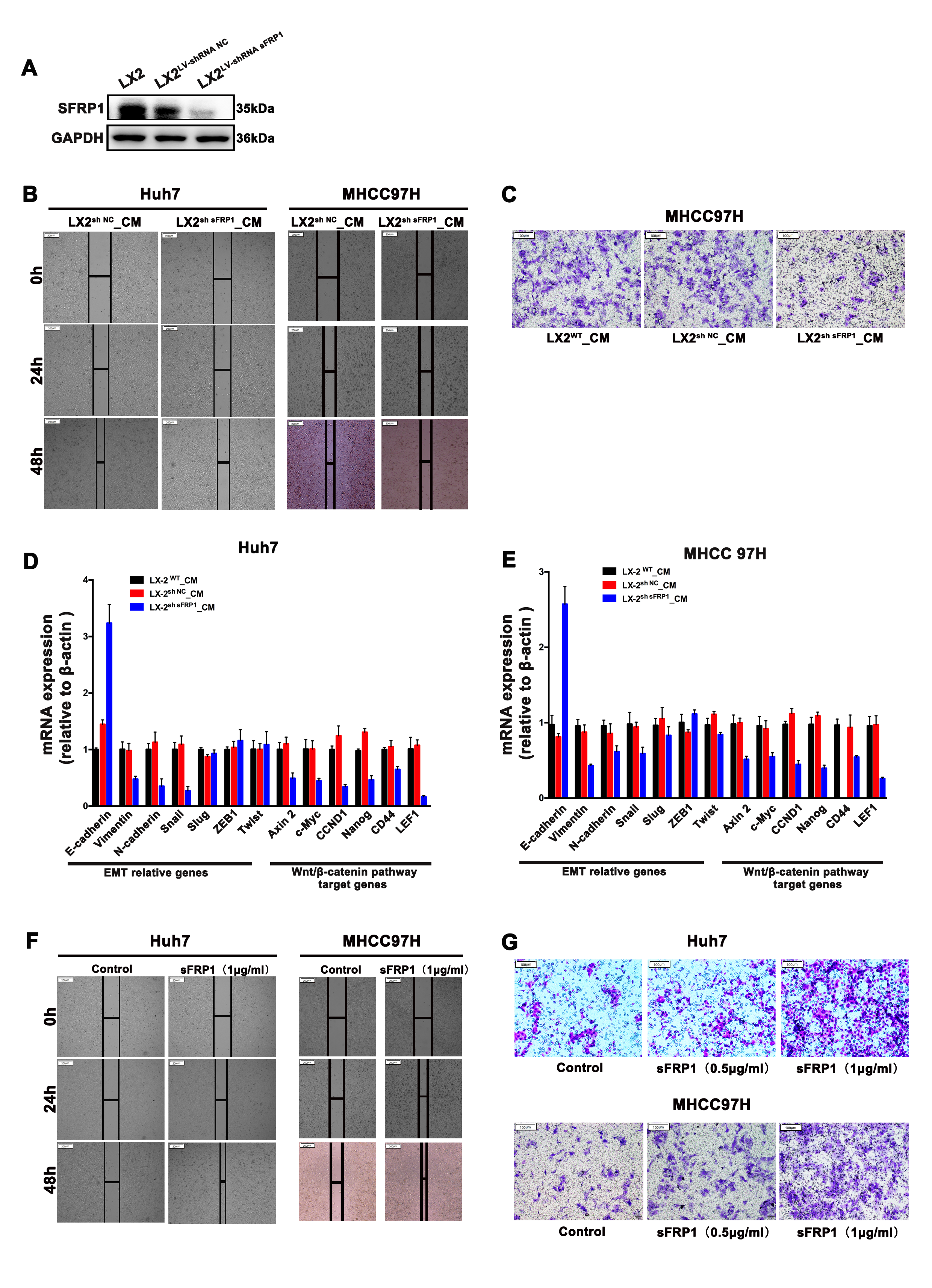

Supplement: Supplementary file 7 — Additional file 7: Figure S5. CM from NE-treated LX-2shRNA sFRP1 cells showed an attenuated promotion of malignant phenotypes of HCC cells in vitro. (A) LX-2 cells transfected with a sFRP1-shRNA lentivirus or a scramble-shRNA lentivirus. The efficiency of sFRP1 knockdown was examined in LX-2shRNA sFRP1 and LX-2shRNA NC cells. (B, C) Compared with CM from NE-treated LX-2shRNA NC, CM from NE-treated LX-2shRNA sFRP1 showed a significant decrease of invasion and migration of HCC cells in vitro, as measured by wound-healing migration assay and Matrigel invasion assay. (D, E) qRT-PCR analyses were used to detect the expression of EMT markers (E-cadherin, N-cadherin, vimentin, snail, slug, ZEB1, and Twist), stemness marker Nanog and target genes of Wnt/β-catenin signaling (Axin2, c-Myc, CCND1, CD44, and LEF1) in Huh7 and MHCC 97H cells exposed to CM from LX-2shRNA sFRP1 versus LX-2shRNA NC versus LX2. (F, G) Exogenous sFRP1 promoted the migration and invasion of HCC cells in vitro, as measured by wound-healing migration assay and Matrigel invasion assay. [file 13046_2020_1568_MOESM7_ESM.tif]

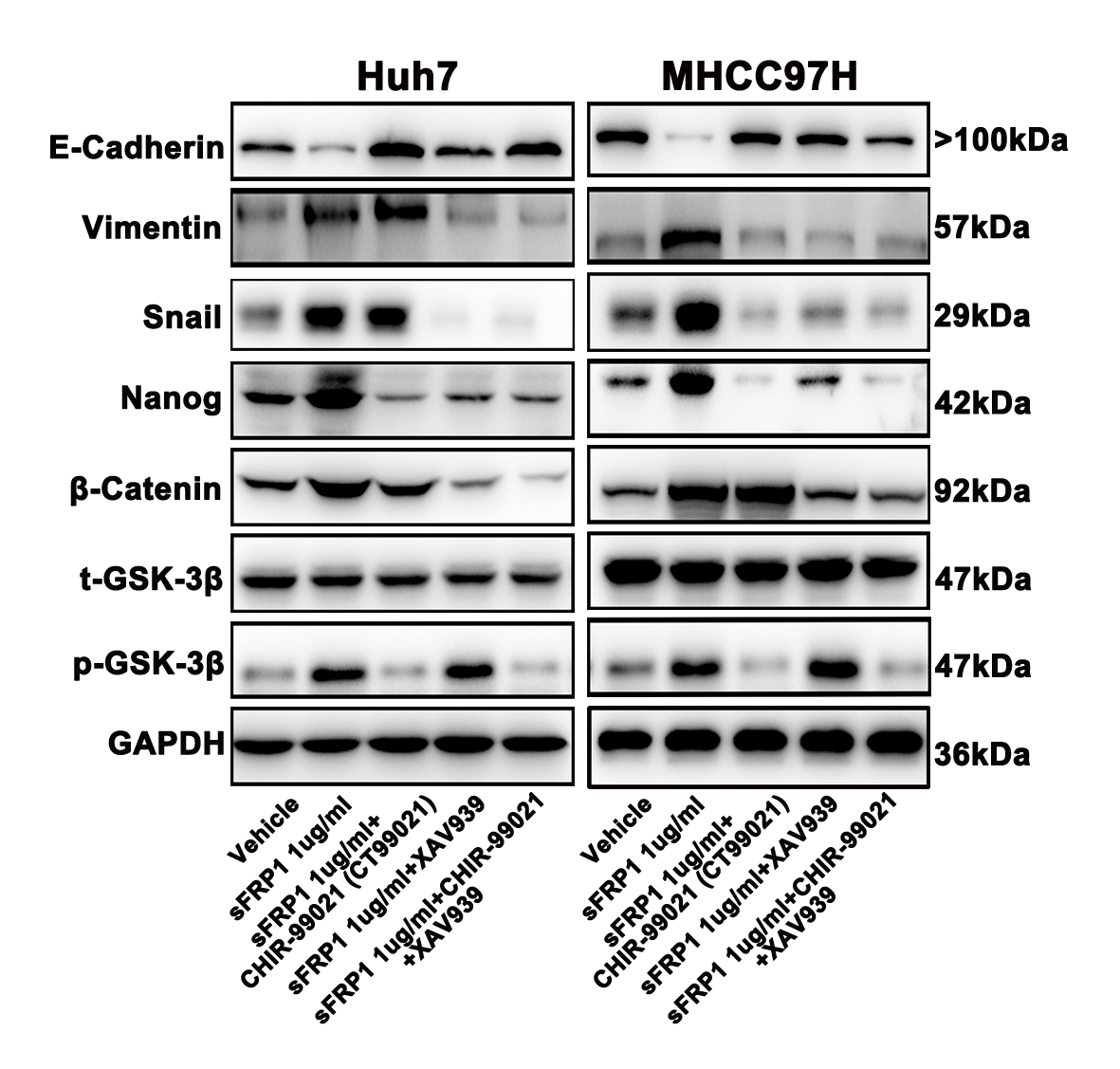

Supplement: Supplementary file 8 — Additional file 8: Figure S6. CHIR 99021 and XAV939 influenced EMT and β-catenin activation induced by sFRP1. [file 13046_2020_1568_MOESM8_ESM.tif]

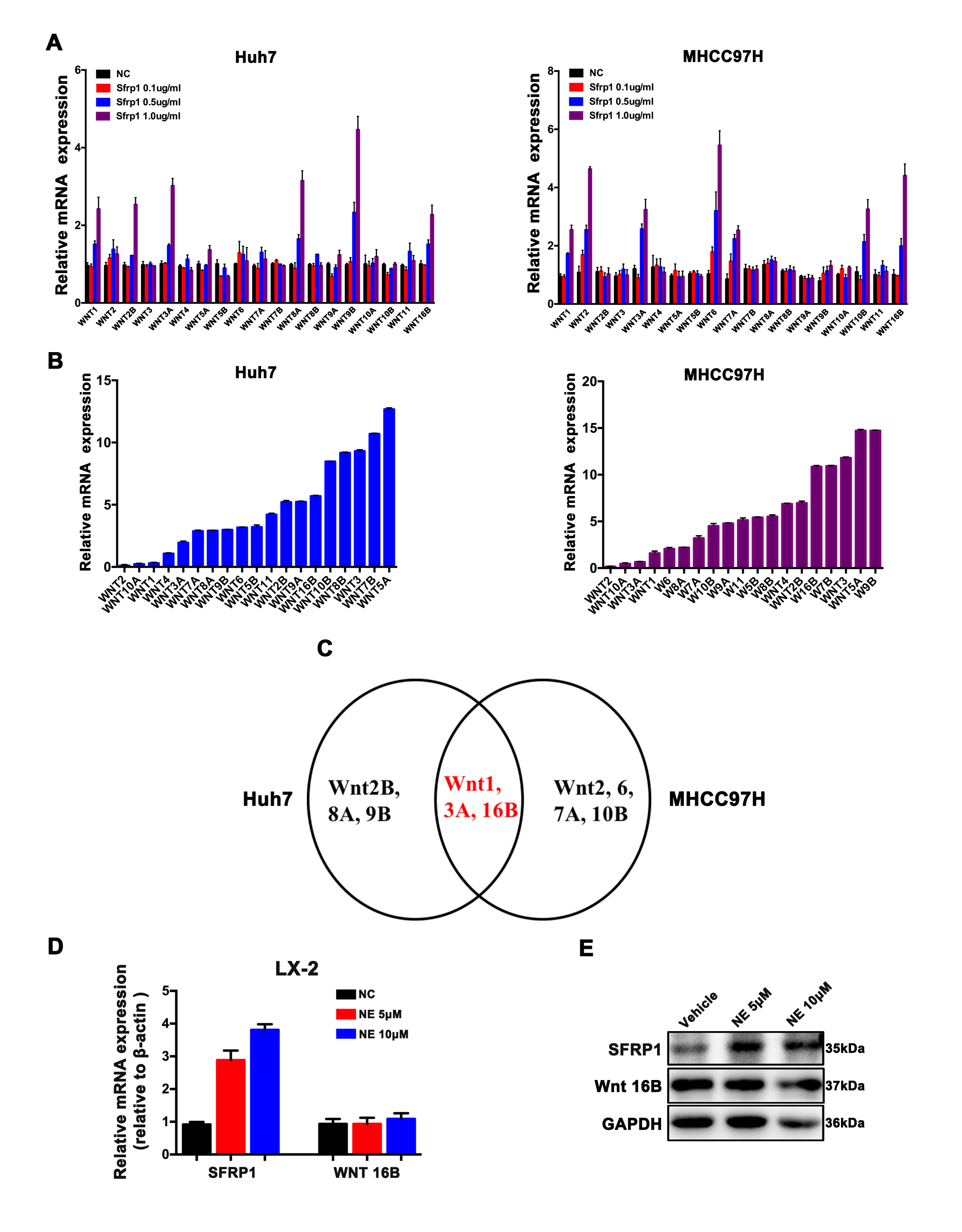

Supplement: Supplementary file 9 — Additional file 9: Figure S7. Expression of Wnt family members in HCC cells exposed to sFRP1. (A, B) qRT-PCR analyses showed the expression levels of 19 Wnt family members in Huh7 cells exposure to 0.1, 0.5, or 1 μg/mL sFRP1 for 24 h. Fold changes represent the extent of relative mRNA change. (C) Wnt1, Wnt3A and Wnt16B were up-regulated in both sFRP1-treated HCC cells (MHCC97H and Huh7 cells). (D, E) There was a significant increase of sFRP1 in LX-2 cells treated with NE (0, 5, and 10 μM) whereas no significant change of Wnt16B was observed, as detected by qRT-PCR and western blot. [file 13046_2020_1568_MOESM9_ESM.tif]

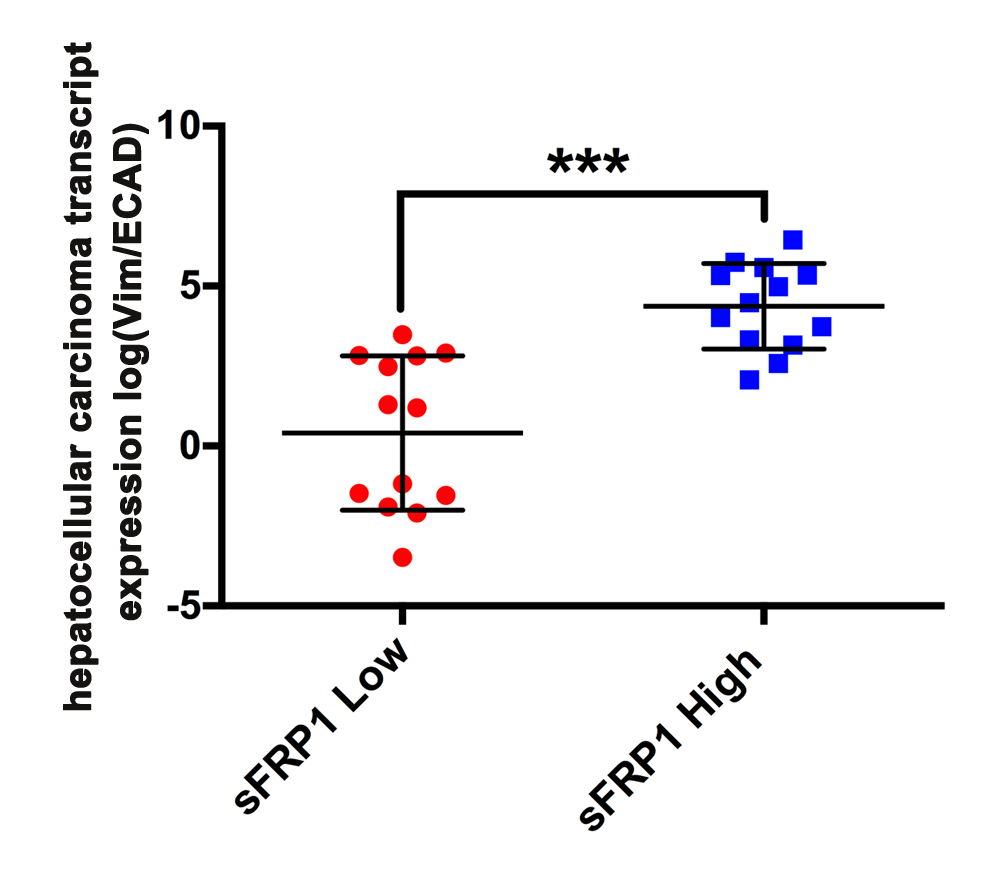

Supplement: Supplementary file 10 — Additional file 10: Figure S8. sFRP1 expression in non-tumoral tissues associated with EMT in HCC. Taken the median mRNA expression level of sFRP1 in non-tumoral tissues as a threshold, we classified the cases into two groups, a low sFRP1 group and a high sFRP1 group. Using the ratio of the mRNA expression of Vimentin and E-cadherin (Vimentin /E-cadherin) as an indicator of EMT, Vimentin /E-cadherin ratio in HCC tissues was significantly upregulated in the high sFRP1 group. [file 13046_2020_1568_MOESM10_ESM.tif]
