# Supplementary material for: Norepinephrine-stimulated HSCs secrete sFRP1 to promote HCC progression following chronic stress via augmentation of a Wnt16B/β-catenin positive feedback loop
Source: J Exp Clin Cancer Res. 2020 Apr 15;39:64. doi: 10.1186/s13046-020-01568-0 (PMC7158101; doi:10.1186/s13046-020-01568-0)
Supplement: Supplementary file 5 — Additional file 5: Table S2. A total of 31 differentially expressed genes were identified in NE-treated versus vehicle-treated LX-2 cells. [file 13046_2020_1568_MOESM5_ESM.docx]

**Table S2. A total of 31 differentially expressed genes were identified in NE-treated *versus* vehicle-treated LX-2 cells**

| **Gene Symbol** | **Description** | **Probe_Set_ID** | | **Fold change** |
| --- | --- | --- | --- | --- |
| SMAD5  COL11A1 | SMAD family member 5  collagen, type XI, alpha 1 | | 205187_at  229271_x_at | 19.67  19.10 |
| CCNT2 | cyclin T2 | | 213743_at | 18.73 |
| CD200 | CD200 molecule | | 209583_s_at | 15.14 |
| FGFR1OP2 | FGFR1 oncogene partner 2 | | 1556282_at | 11.13 |
| PTGER3 | prostaglandin E receptor 3 (subtype EP3) | | 210375_at | 9.58 |
| CDH19 | cadherin 19, type 2 | | 1555403_a_at | 8.64 |
| ADAMTS6 | ADAM metallopeptidase with thrombospondin type 1 motif, 6 | | 1570351_at | 7.39 |
| ADAM17  COL4A1  HIF1AN  GFRA1  CD209  EGFR  TMEM121  TMEM33  SFRP1  ADAMTS10  ADAMDEC1  TMEM251  COX20  TMEM165  CDK12  CD53  CDC42SE1  ADAM18  COL1A1  HSPA9  ADAM6  FGF14-AS2  COL3A1 | ADAM metallopeptidase domain 17  collagen, type IV, alpha 1  hypoxia inducible factor 1, alpha  subunit inhibitor  GDNF family receptor alpha 1  CD209 molecule  epidermal growth factor receptor  transmembrane protein 121  transmembrane protein 33  secreted frizzled-related protein 1  ADAM metallopeptidase with thrombospondin type 1 motif, 10  ADAM-like, decysin 1  transmembrane protein 251  COX20 cytochrome C oxidase assembly factor  transmembrane protein 165  cyclin-dependent kinase 12  CD53 molecule  CDC42 small effector 1  ADAM metallopeptidase domain 18  collagen, type III, alpha 1  heat shock 70kDa protein 9 (mortalin)  ADAM metallopeptidase domain 6, pseudogene  FGF14 antisense RNA 2  collagen, type II, alpha 1 | | 213532_at  211980_at  226648_at    205696_s_at  1555729_a_at  1565484_x_at  219663_s_at  235907_at  202036_s_at  230341_x_at  206134_at  213246_at  224824_at  230657_at  219226_at  242946_at  222537_s_at  207597_at  201852_x_at  232200_at  237909_at  230351_at  213492_at | 6.40  5.27  5.21  3.80  3.78  3.66  3.36  3.35  3.34  3.29  3.25  3.10  2.74  2.58  2.46  2.42  2.37  2.34  2.25  2.22  2.20  2.15  2.04 |
